# Gene-trait matching among *Bifidobacterium dentium* strains reveals various glycan metabolism loci including a strain-specific fucosyllactose utilization cluster

**DOI:** 10.3389/fmicb.2025.1584694

**Published:** 2025-05-12

**Authors:** Ortensia Catalano Gonzaga, Stephen McKenna, Ian O’Neill, Paul D. Cotter, Fionnuala M. McAuliffe, Aidan Coffey, Douwe van Sinderen, Francesca Bottacini

**Affiliations:** ^1^APC Microbiome Ireland, University College Cork, Cork, Ireland; ^2^School of Microbiology, University College Cork, Cork, Ireland; ^3^Department of Biological Sciences, Munster Technological University, Cork, Ireland; ^4^Food Biosciences, Teagasc Food Research Centre Moorepark, Cork, Ireland; ^5^UCD Perinatal Research Centre, School of Medicine, University College Dublin, National Maternity Hospital, Dublin, Ireland

**Keywords:** *Bifidobacterium*, HMO, pangenome, comparative genomics, GIT

## Abstract

In contrast to other human-associated bifidobacteria, *Bifidobacterium dentium* is commonly classified as an opportunistic pathogen as its presence in the oral cavity has been associated with the development of dental caries. While *B. dentium* is frequently isolated from the oral cavity of children with caries, recent microbiome investigations and preliminary genomic analyses have suggested that this species is also adapted to colonize the gastrointestinal tract. Understanding the genetic and metabolic adaptations that enable this flexible colonization ability is crucial to clarify its role in human health and disease. To assess *B. dentium* genomic diversity and metabolic potential, the current study presents analysis and characterization of 10 complete genome sequences from recently isolated *B. dentium* strains obtained from human fecal samples together with 48 publicly available genome sequences. We investigated genetic loci predicted to be involved in host interaction and carbohydrate utilization in this species by means of comparative genomics, pan-genome analysis, and gene-trait matching. These analyses identified gene clusters involved in the utilization of plant-derived glycans and, for the first time, revealed *B. dentium* strains capable of utilizing human milk oligosaccharides (HMOs) through a fucosyllactose utilization cluster homologous to the one found in several infant-derived bifidobacterial species. Moreover, additional investigations of strain-specific genetic features highlighted a taxon that is evolved to colonize multiple niches and to compete with other colonizers. These findings challenge the narrow classification of *B. dentium* as an opportunist and underscore its ecological versatility.

## 1 Introduction

Various members of the genus *Bifidobacterium* are common commensals of the human gastrointestinal tract, representing Gram-positive, anaerobic, non-motile, non-spore forming, high GC-content bacteria, being members of the *Bifidobacteriaceae* family, and belonging to the *Actinomycetota* phylum (Turroni et al., 2011; Lugli et al., 2020a; Hidalgo-Cantabrana et al., 2017). Over 100 distinct (sub)species are currently recognized as taxonomic members of the *Bifidobacterium* genus (https://lpsn.dsmz.de/genus/bifidobacterium), with this number still increasing every year (Alessandri et al., 2021). *Bifidobacterium* is the predominant commensal genus found in the infant gut microbiota, and this remarkable dominance appears to be supported by the infant’s exclusively milk-based diet (Turroni et al., 2012). Following weaning, the relative abundance of *Bifidobacterium* in the human gut microbiota decreases to and stabilizes at 3–6% in adults, with a further drop in the elderly (O’Callaghan and van Sinderen, 2016; Lugli et al., 2020a). The presence of bifidobacteria in the human gut has been associated with various host health benefits, such as immune system development, production of vitamins and short-chain fatty acids, alleviation of gut disorders, and inhibition of enteropathogens (Wong et al., 2020; O’Callaghan and van Sinderen, 2016; Engevik et al., 2021a). Bifidobacteria have also been reported to elicit a positive impact on the overall human gut physiology, for example through the degradation of complex carbohydrates that are indigestible to the host (Duranti et al., 2020; Turroni et al., 2018). Specific bifidobacterial strains and species possess the ability to degrade host-derived glycans such as mucin O-glycans and human milk oligosaccharides (HMOs), but also various dietary glycans such as plant-derived poly- and oligo-saccharides, thus expanding the metabolic abilities of their host (Duranti et al., 2020; Turroni et al., 2018).

*Bifidobacterium dentium* is listed as an opportunistic pathogen due to its association with dental caries (Kaur et al., 2013; Kressirer et al., 2018). Correlations have been established between the presence of oral bifidobacteria, including *B. dentium*, and occlusal caries lesions in children and adults (Manome et al., 2019; Spatafora et al., 2024). Nonetheless, its overall impact on human health is unclear, as some *B. dentium* strains have been reported to exert potential beneficial host effects (Engevik et al., 2021a). Investigations have highlighted the presence of *B. dentium* in healthy infant stool, and as having a relative abundance of 0.7% in healthy human adults (Duranti et al., 2017; Engevik et al., 2021a). Moreover, *B. dentium* has been reported to alleviate endoplasmic reticulum stress, minimize inflammation, provide a protective effect on the intestinal barrier and modulate visceral pain in the intestine (Pokusaeva et al., 2017; Engevik et al., 2021b; Zhao et al., 2021).

Investigation of the metabolic capabilities of *B. dentium* has so far revealed a greater metabolic versatility when compared to other bifidobacteria. *In silico* genomic screening of this taxon predicted genes belonging to members of 29 glycoside hydrolase (GH) families, displaying an extensive digestive ability toward a wide range of carbohydrates with a core glycobiome focused on the degradation of plant-derived glycans and simple carbohydrates (Lugli et al., 2020a; Engevik et al., 2021a). *B. dentium* genomes harbor genes predicted to encode enzymes involved in HMO degradation, such as putative β-galactosidases, α-L-fucosidases and sialidases, although it appears that members of this species do not directly access such carbohydrates since no growth was observed on HMOs (Lugli et al., 2020b; Moya-Gonzálvez et al., 2021). This GH reservoir coupled with a high GH index, which is the normalization of GH count based on genome size, would be consistent with the presumed ability of *B. dentium* to colonize different niches within the host (Milani et al., 2015; Lugli et al., 2020a).

All bifidobacteria including *B. dentium* have been shown to use non-specific hydrophobicity and electrostatic forces to adhere to host tissues (González-Rodríguez et al., 2013; Westermann et al., 2016). Furthermore, most members of the *Bifidobacterium* genus harbor gene clusters encoding biosynthetic abilities for the production of type VIa, tight adherence (Tad) and/or sortase-dependent pili, which have been implicated in host cell adhesion and gut colonization (Turroni et al., 2013; O’Connell Motherway et al., 2011). Of note, bifidobacterial genomes can harbor multiple pilus-biosynthesis loci, with *B. dentium* Bd1 predicted to possess up to seven gene clusters (Westermann et al., 2016; Penno et al., 2022; Foroni et al., 2011). Additionally, as has been shown for other bifidobacteria, *B. dentium* may use extracellular polysaccharides to adhere to host tissues (polysaccharides secreted in the extracellular matrix or loosely associated to the cell surface; Fanning et al., 2012), although their role in adhesion has not been explored in this species (Bottacini et al., 2014; Westermann et al., 2016).

Although some aspects regarding *B. dentium* as a member of the intestinal microbiota have been investigated, an extensive and in-depth analysis of these features, with an emphasis on host interaction and gut colonization, has to the best of our knowledge not yet been conducted. To gain a better understanding of *B. dentium* genomic diversity and metabolic potential we present here the analysis and characterization of complete genome sequences of 10 recently isolated *B. dentium* strains. Through a combined genotypic and phenotypic characterization in terms of their carbohydrate metabolism we were able to identify in this species a fucosyllactose utilization cluster homologous to a gene cluster identified in several infant-derived bifidobacterial species, as well as gene clusters known in other bifidobacteria to be involved in carbohydrate utilization (e.g., raffinose, sucrose, xylooligosaccharides (XOS), xylose, mannitol). Moreover, we investigated, through a comparative genomic analysis, the diversity of genetic loci involved in host interaction and colonization.

## 2 Materials and methods

### 2.1 *Bifidobacterium dentium* DNA isolation, sequencing, and assembly

Bifidobacterial overnight cultures were obtained by cultivating strains in modified De Man-Rogosa-Sharpe (mMRS) medium prepared from first principles (De Man et al., 1960) supplemented with 0.05% cysteine-HCl (Sigma Aldrich, Steinheim, Germany) and 1% lactose at 37°C under anaerobic conditions in a modular atmosphere-controlled system (Don Whiteley Scientific, West Yorkshire, UK). Following growth, total bifidobacterial DNA was obtained as described previously (Bottacini et al., 2018). Genome sequencing of *B. dentium* strains was performed commercially by the Norwegian Sequencing Centre (NSC), located at the University of Oslo using a Pacific Biosciences Sequel II system. Following sequencing, raw reads were *de novo* assembled into complete, i.e., circular, chromosomes using Flye assembler v 2.9.1 (https://github.com/fenderglass/Flye; Freire et al., 2022) and employing default parameters. Following read assembly, the obtained complete chromosome sequences underwent Open Reading Frame (ORF) prediction and automatic annotation using Prokka (v1.14.6; Seemann, 2014) available in the Galaxy online platform (Afgan et al., 2018) and employing default parameters. Functional assignment of identified ORFs was performed employing a reference protein database containing all *Bifidobacterium* RefSeq proteins from publicly available complete genomes (Cuccuru et al., 2014; Seemann, 2014). For functional assignment, an *e*-value cut-off of <0.0001 in BLASTP alignments (Altschul et al., 1990) was used within Prokka (v1.14.6; Seemann, 2014). Following annotation, circular contigs were rearranged to start at the *dnaA* gene using Circlator (v1.5.5; Hunt et al., 2015). GenBank editing and manual inspection were performed using Artemis v18 (http://www.sanger.ac.uk/resources/soft-ware/artemis/; Rutherford et al., 2000).

### 2.2 Comparative and pan-genome analysis of *Bifidobacterium dentium*

Five complete and thirty-three draft *B. dentium* genome sequences were obtained from the National Center for Biotechnology Information (NCBI) (Sayers et al., 2022; Lugli et al., 2020a) and combined with the 10 complete genomes of *B. dentium* strains sequenced here in order to perform a comparative genome analysis. Calculation of the *B. dentium* pangenome was conducted on this total of 48 *B. dentium* genomes using the pangenome pipeline Roary v3.13.0 (Galaxy version 4.0.0rc1) using default settings (Page et al., 2015) and then visualized using R v4.5.0 (https://www.r-project.org; R Core Team, 2021).

The presence of mobile elements and genes encoding transposases or integrases in the available (i.e., 15) complete genomes of *B. dentium* was performed by screening all deduced protein sequences using hmmscan alignments against the PFAM database (http://ftp.ebi.ac.uk/pub/databases/Pfam; Mistry et al., 2021) employing an *e*-value cut-off of <0.0001. The ICEfinder v1.0 online tool (https://bioinfo-mml.sjtu.edu.cn/ICEfinder/index.php; Liu et al., 2019) was employed to identify Integrative Conjugative Elements (ICE) using default settings and the prediction was confirmed by the presence of conjugation-related genes such as relaxases, surface proteins and type IV secretion systems. Identification of putative prophage sequences was performed using VirSorter2 v2.2.4 (Guo et al., 2021) using default settings, while Artemis v.16 (Rutherford et al., 2000) was used to manually inspect genome sequences. The VIRIDIC v1.1 tool (https://rhea.icbm.uni-oldenburg.de/viridic/; Moraru et al., 2020) was employed to calculate and visualize the intergenomic similarities of the predicted prophage sequences employing default settings. The prediction of clustered regulatory interspaced short palindromic repeats (CRISPR) and associated Cas-encoding genes was performed using CRISPRCasFinder v1.1.2 (Couvin et al., 2018) with default settings. Each predicted proteome was screened for the presence of Restriction-Modification (RM) systems using the BLASTP alignment function of the REBASE database (https://rebase.neb.com/rebase/rebase.html; Roberts et al., 2023) with default settings.

The presence of gene clusters encoding genetic functions involved in host interaction (e.g., sortase-dependent pilus biosynthesis clusters and exopolysaccharide biosynthesis clusters) was identified by screening all predicted protein sequences of the 15 complete *B. dentium* genome sequences using hmmscan alignments against the PFAM database (http://ftp.ebi.ac.uk/pub/databases/Pfam; Mistry et al., 2021) employing an *e*-value cut-off of <0.0001. In particular, sortase-dependent pilus biosynthesis clusters were identified by the presence of a predicted sortase-encoding gene flanked by two or more genes predicted to encode a secreted protein with a sortase cleavage domain.

Similarly, extracellular polysaccharide (EPS) biosynthesis gene clusters were predicted based on the presence of clustered genes encoding functions involved in EPS biosynthesis, such as glycosyltransferases, a flippase or an ABC transporter, a priming glycosyltransferase and a chain-length regulator (Bottacini et al., 2018).

The *in silico* prediction of gene clusters involved in bacteriocin biosynthesis, export and immunity was performed using the BAGEL4 tool (Bacteriocin Genome minimal tool, http://bagel4.molgenrug.nl/; van Heel et al., 2018), which identifies clustered genes related to bacteriocin production (van Heel et al., 2018). ORFs of a given identified bacteriocin cluster were analyzed using Artemis v.16 (Rutherford et al., 2000), and protein functions were predicted by searching for homologs using BLASTP (Altschul et al., 1990) against the non-redundant (nr) NCBI database using default settings. In addition, prediction of the signal peptide and cleavage site of the candidate bacteriocin-encoding gene was performed on the coding sequence using the SignalP v5.0 server with default settings (Bendtsen et al., 2004; https://services.healthtech.dtu.dk/services/SignalP-5.0/). Furthermore, putative transmembrane domains were predicted with the TMHMM v2.0 server using default settings (Krogh et al., 2001; https://services.healthtech.dtu.dk/services/TMHMM-2.0/).

Screening and prediction of glycoside hydrolase (GH)-encoding genes in 10 *B. dentium* genomes sequenced within this study were performed using dbCAN2 tool using DIAMOND v3 and HMMER v3.4 alignments and default settings (Zhang et al., 2018).

### 2.3 Comparative analysis of the 2′-fucosyllactose/3-fucosyllactose utilization locus

Complete genome sequences of *Bifidobacterium longum* subsp. *infantis* ATCC 15697, *Bifidobacterium longum* subsp*. iuvenis* JDM301, *Bifidobacterium catenulatum* subsp. *kashiwanohense* APCKJ1, and *Bifidobacterium pseudocatenulatum* DSM 20438, obtained from the National Center for Biotechnology Information (NCBI), and those of *Bifidobacterium dentium* MB0185 and MB0224, sequenced as part of the current study, were used for a linear comparison of the predicted or experimentally proven fucosyllactose metabolism cluster using Easyfig v2.2.5 with default settings (James et al., 2019; Sullivan et al., 2011). For phylogenetic analysis of the predicted FumA1 and FumA2 homologs encoded by *B. dentium* MB0185 and MB0224 (these were specifically named FumA1_bd185_ and FumA2_bd185_, and FumA1_bd224_ and FumA2_bd224_, respectively), the protein sequences of seventy-two previously characterized/predicted α-fucosidases (James et al., 2019) were aligned to these predicted α-fucosidases using CLUSTALW v2.1 using default settings (Thompson et al., 2002). The Maximum Likelihood phylogenetic tree was built using MEGAX v12 software tool (https://www.megasoftware.net; Kumar et al., 2024) and visualized using iTOL v7.1 (Letunic and Bork, 2021) using default settings. The *E. coli* K-12 DnaA protein was used as an outgroup for phylogenetic inference.

### 2.4 *Bifidobacterium dentium* carbohydrate-dependent growth profiles

Growth profiles of the 10 newly sequenced *B. dentium* strains were tested on 34 distinct carbohydrates as individual and sole carbon sources (Supplementary Table 1). Briefly, a 10% (w/v) stock solution of each carbohydrate was prepared using distilled water, filter-sterilized using a 0.2 μm membrane filter, and then stored at 4°C until required. Growth medium was prepared using modified de Man Rogosa Sharpe (mMRS) medium supplemented with 0.5% (v/v) of an individual carbohydrate stock solution and 0.05% of L-cysteine-HCl (Watson et al., 2013). mMRS supplemented with 0.05% (v/v) cysteine-HCl but without any carbohydrate served as the negative control, while mMRS supplemented with 0.05% (v/v) cysteine-HCl and 0.5% (v/v) lactose served as a positive control. To obtain growth profiles, *B. dentium* overnight cultures were diluted to optical density at 600 nm (OD_600nm_) of 0.05 with growth medium described above. Cultures were then grown anaerobically at 37°C in technical duplicates and the OD_600nm_ was measured manually after 24 h, using a UV-1280 UV–VIS spectrophotometer (Shimadzu Corporation, Kyoto, Japan). The reported OD_600nm_ values were expressed as the mean of duplicate measurements and a minimum of two independent growth experiments were performed for each carbohydrate. Growth of bacterial strains was assessed based on OD_600nm_ values, where an OD_600nm_ value equal to or larger than 0.4 was designated as the cut-off point for good growth, with OD_600nm_ values between 0.2 and 0.4 as intermediate growth and below 0.2 classified as poor/no growth (Bottacini et al., 2018).

In order to obtain a more detailed analysis of growth profiles/behavior of selected *B. dentium* strains when cultivated on particular carbohydrates, growth curves were performed in mMRS supplemented with a particular carbohydrate as the sole carbon/energy source using an absorbance 96 plate reader (Enzo Life Sciences, Inc., United States) which measured optical density at 620 nm (fixed wavelength setting) every hour for 45 h. To acquire more precise OD measurements during the initial hours of growth, any remaining lactose present in the overnight medium was removed by two washing steps with Phosphate Buffered Saline (PBS). Subsequently, the cell cultures were harvested by centrifugation at 3,800 × g for 5 min and resuspended in 1 mL of carbohydrate-free mMRS. Cells were then diluted to OD_600nm_ of 0.05 and inoculated into mMRS containing 0.05% (v/v) L-cysteine and 0.5% (v/v) of 2′-fucosyllactose (2’-FL), 3-fucosyllactose (3-FL), difucosyllactose (DFL), lacto-N-tetraose (LNT), lacto-N-neotetraose (LNnT), 3′-sialyllactose (3’-SL), 6′-sialyllacose (6’-SL) or no carbohydrate, the latter acting as a negative control. Cultures were incubated anaerobically at 37°C for 45 h. *B. breve* UCC2003 was used as a positive control for growth on LNT and LNnT, and as a negative control for growth on 2’-FL, 3-FL, DFL, 6’-SL and 3’-SL.

### 2.5 Plasmid constructions for protein overproduction and purification

The construction of plasmids pET28b-FumA1_bd185_ and pET28b-FumA2_bd185_ (which contain the genes encoding the predicted GH95 and GH29 family α-fucosidases, respectively, of *B. dentium* MB0185) was performed according to a previously established method (James et al., 2019). DNA fragments encompassing the predicted fucosidase-encoding genes, *fumA1_bd185_* (corresponding to the *B. dentium* MB0185 genome locus tag MB0185_2106) or *fumA2_bd185_* (corresponding to the *B. dentium* MB0185 genome locus tag MB0185_2105) were PCR generated employing chromosomal DNA of *B. dentium* MB0185 as a template and using Q5 High-Fidelity DNA polymerase and primer combinations GH95F and GH95R, or GH29BF and GH29BR, respectively (Supplementary Table 2). The two generated amplicons were restricted with NotI and NheI, or EcoRI and NheI, respectively, and then ligated into the NotI and NheI, or EcoRI and NheI-digested plasmid pET28b, which harbors an N-terminal His6-encoding sequence to facilitate downstream protein purification (Nilsson et al., 1997). Ligation mixtures were introduced into *E. coli* BL21 by electrotransformation and transformants were then selected based on kanamycin resistance. Transformants were checked for plasmid content using colony PCR and the integrity of positively identified clones was verified by sequencing, performed at Genewiz (Leipzig, Germany).

Protein overproduction was obtained using NZY auto-induction LB medium (NZYTech, Lisboa, Portugal). In brief, 100 mL of NZY Auto-Induction LB medium supplemented with 100 μg/mL kanamycin was inoculated in a baffled flask with a 1% inoculum of a pre-culture grown aerobically overnight at 37°C, followed by incubation at 24°C in an orbital incubator at 300 rpm. After 24 h cells were harvested by centrifugation, washed, and concentrated in lysis buffer (50 mM NaH_2_PO_4_, 300 mM NaCl, 10 mM imidazole; pH 7.5) (Friess et al., 2024). Cell extracts were prepared using 106 mm glass beads and the mini-bead-beater-8 cell disrupter (Biospec Products, Bartville, Oklahoma, United States). After homogenization, the glass beads and cell debris were removed by centrifugation, while the supernatant containing the cytoplasmic fractions was retained. Protein purification from the cytoplasmic fraction was performed using Ni-NTA matrices in accordance with the manufacturer’s instructions (Qiagen). Elution fractions were analyzed by SDS polyacrylamide (12.5%) gel electrophoresis. Following electrophoresis, gels were fixed and stained with Commassie Brilliant blue to identify fractions containing the purified protein. Rainbow prestained low molecular weight protein markers (New England Biolabs, Herdfordshire, UK) were used to estimate the molecular weight of the purified proteins. Elution fractions of interest were then concentrated and dialyzed in 20 mM sodium phosphate buffer pH7 using Amicon® Ultra Filters Merck Millipore (Merck, Darmstadt, Germany). The concentration of the protein was then estimated using the Qubit^®^ Fluorometer, assuming an estimated purity exceeding 95% as based on visual inspection, and the corresponding protein assay kit (Thermo Scientific, Gloucester, UK).

### 2.6 Hydrolytic enzyme activity assays and HPAEC-PAD analysis

Assays to determine the hydrolytic activities specified by the protein products of the predicted fucosidase-encoding genes *fumA1_bd185_* (corresponding to *B. dentium* MB0185_2106) and *fumA2_bd185_* (corresponding to *B. dentium* MB0185_2105) were performed using 2’-FL, 3-FL, DFL, lacto-N-fucopentaose I (LNFPI), lacto-N-fucopentaose II (LNFPII) or lacto-N-fucopentaose III (LNFPIII) as substrates. Briefly, each purified protein was added (at a final concentration of 0.025 mg/mL) to 20 mM morpholinepropanesulfonic acid (MOPS) (pH 7.0) buffer and 1 mg/mL (wt/vol) of one of the above-mentioned sugars in a final volume of 1 mL, followed by overnight incubation at 37°C.

HPAEC-PAD analysis was performed using a Dionex (Sunnyvale, CA) ICS-3000 system equipped with a CarboPac PA1 analytical-exchange column (dimensions, 250 mm by 4 mm) with a CarboPac PA1 guard column (dimensions, 50 mm by 4 mm) and a pulsed electrochemical detector (ED40) in PAD mode (Dionex). Samples were eluted at a constant flow rate of 1.0 mL/min at 30°C using Milli-Q water. Chromatographic profiles of 1 mg/mL (wt/vol) of 2’-FL, 3-FL, DFL, LNFPI, LNFPII and LNFPIII dissolved in Milli-Q water were used as references. The obtained chromatogram profiles were integrated and evaluated using Chromeleon software (version 6.70; Dionex Corporation).

### 2.7 Protein structure prediction

For the prediction of the three-dimensional structure of FumA1_bd185_, FumA2_bd185_ and *B. dentium* MB0148 propionicin-SM1-like bacteriocin, sequences in FASTA format were used as inputs for AlphaFold2-multimer-v2 (AFm) implemented in ColabFold v1.5.2 with MMseqs2 (Jumper et al., 2021; Mirdita et al., 2022) using default settings. The PAE plot for the best mode was generated using PAE Viewer with default settings (Elfmann and Stülke, 2023).

### 2.8 Data visualization

Data relating to the pan-genome and GHs was processed and illustrated graphically using the stats, reshape, gridtext, gridExtra, tidyverse, gplots, ggplot2, and pheatmap packages in R v4.5.0 (https://www.r-project.org; R Core Team, 2021). Linear comparisons between the genomic loci predicted to be involved in fucosyllactose utilization, exopolysaccharide biosynthesis, or bacteriocin production were generated with Easyfig v2.2.5 (Sullivan et al., 2011). Phylogenetic trees were visualized using the Interactive Tree of Life (iTOL) tool v7.1 (Letunic and Bork, 2021). Visualization of intergenomic similarities of the predicted prophage sequences was performed using the VIRIDIC v1.1 tool with default settings (https://rhea.icbm.uni-oldenburg.de/viridic/; Moraru et al., 2020). Amino acid sequence alignments were generated with ClustalOmega using default settings (https://www.ebi.ac.uk/Tools/msa/clustalo/; Sievers and Higgins, 2014). Graphical views of genomes were generated using Mauve v2.4.0 (Darling et al., 2004) and DNAplotter v18.2.0 (Carver et al., 2009) using default settings. The three-dimensional structures obtained from the AlphaFold2 default algorithm were visually inspected by ChimeraX v1.8 (Pettersen et al., 2021). Alignments of predicted three-dimensional structures were performed using the *MatchMaker* tool implemented in the Chimera software v1.2.5 using default settings (Meng et al., 2006).

### 2.9 Sequence data deposition

The 10 genomes of *B. dentium* strains that were sequenced to completion in the current study have been deposited in the NCBI repository under the BioProject accession number PRJNA1073879. The accession numbers corresponding to each of the ten sequenced genomes are listed in Table 1.

**Table 1:** General genome features of *B. dentium* strains.

Genomes	Isolation source	Genome length (bp)	Contigs	ORFs	GHs number	GH index (%)	tRNA	GC content (%)	Accession number
*B. dentium* Bd1	Dental caries	2,636,370	1	2,104	81	3.8	56	58.5	NC_013714
*B. dentium* JCM1195	Dental caries	2,635,669	1	2,099	81	3.9	56	58.5	AP012326
*B. dentium* N8	Infant fecal sample	2,535,489	1	2,030	–	–	56	58.5	NZ_CP072502
*B. dentium* E7	Infant fecal sample	2,552,511	1	2,017	–	–	55	58.3	NZ_CP072503
*B. dentium* NCTC11816	Dental caries	2,635,828	1	2,099	81	3.9	56	58.5	NZ_LR134349
*B. dentium* MM0074	Mother fecal sample	2,658,724	1	2,237	82	3.7	54	58.9	CP162927
*B. dentium* MB0076	Infant fecal sample	2,758,140	1	2,335	87	3.7	58	58.5	CP162919
*B. dentium* MB0114	Infant fecal sample	2,634,020	1	2,147	85	4.0	57	58.5	CP162920
*B. dentium* MB0148	Infant fecal sample	2,671,532	1	2,193	84	3.8	55	58.5	CP162921
*B. dentium* MM0176	Mother fecal sample	2,616,313	1	2,161	77	3.6	56	58.5	CP162928
*B. dentium* MB0185	Infant fecal sample	2,729,428	1	2,318	91	3.9	55	58.5	CP162922
*B. dentium* MB0224	Infant fecal sample	2,749,478	1	2,262	89	3.9	56	58.5	CP162923
*B. dentium* MB0372	Infant fecal sample	2,550,034	1	2,033	85	4.2	55	58.5	CP162924
*B. dentium* MB0385	Infant fecal sample	2,654,302	1	2,183	84	3.8	56	58.6	CP162925
*B. dentium* MB0477	Infant fecal sample	2,580,405	1	2,099	77	3.7	56	58.6	CP162926

## 3 Results

### 3.1 Genomic overview of the ten newly sequenced *Bifidobacterium dentium* genomes

The ten *B. dentium* strains used in this study had recently been isolated from fecal samples obtained from mother-infant dyads collected during the MicrobeMom study (Feehily et al., 2023; Moore et al., 2023). The obtained isolates underwent full genome sequencing employing a Pacbio SMRT SEQUEL platform to supplement the previously obtained short-read Illumina sequences, which upon assembly resulted in the generation of a complete (i.e., circular) chromosome (Table 1). The obtained genomes were compared against each other and against five complete and publicly available *B. dentium* genomes. The average genome length of 2,639,883 bp is substantially larger than other human-derived bifidobacterial genomes (Table 1; Duranti et al., 2015; Duranti et al., 2016; Bottacini et al., 2018). The GC% was shown to be consistent across the strains with an average of 58.5% for this species, which is consistent with what previously has been observed for *B. dentium* and for other members of the genus *Bifidobacterium* (Lugli et al., 2020a; Milani et al., 2014). Overall, the number of predicted open reading frames (ORFs) averaged at 2,147 (Table 1), which is consistent with a previous report (Lugli et al., 2020a).

Whole genome sequence alignment performed using Mauve v2.4.0 (Darling et al., 2004; Supplementary Figure 1A) revealed that the *B. dentium* MB0185 genome seems to have undergone a large chromosomal rearrangement of approximately 1,200 Kb through a genome inversion around the origin-terminus axis of the genome, causing a shift in the GC skew [the (G-C)/(G + C) value] (Supplementary Figure 1B). A large chromosomal rearrangement of approximately 2,000 Kb also seems to have occurred in the *B. dentium* MB0224 genome at approximate position 400,000 bp (Supplementary Figure 1A) around the replication terminus region, though in this case the GC skew is preserved (Supplementary Figure 1B).

### 3.2 Pan-genome of *Bifidobacterium dentium* species

**Figure 1:**
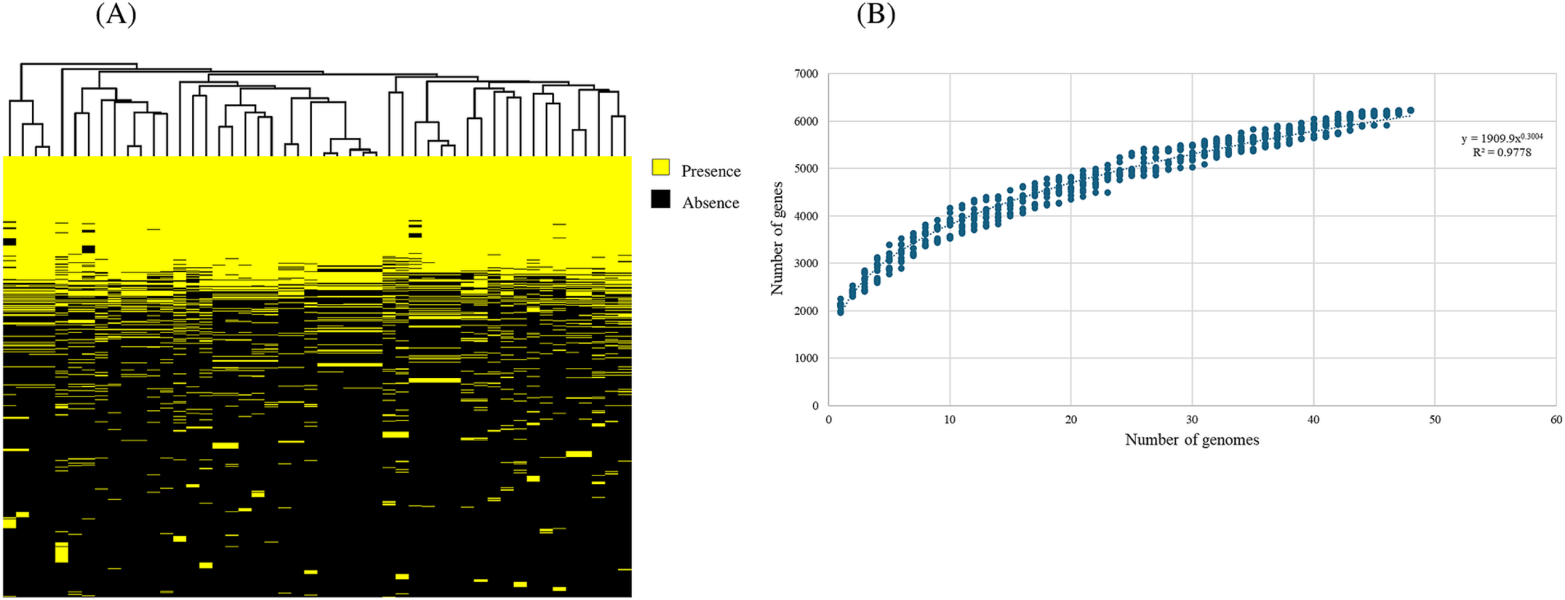
Comparative genomics of 48 *Bifidobacterium dentium* representatives, consisting of 38 publicly available genomes as well as 10 strains sequenced as part of this study. **(A)** Binary map representing two-way hierarchical clustering analysis (HCL) conducted on the 48 *B. dentium* genomes. Presence of genes is indicated in yellow, while the absence of genes is shown in black. The relationship between the genomes is represented by the clustering above the binary map performed through R. **(B)** Pan-genome analysis of *B. dentium* species. The curve represents the variation in pan-genome size as additional genomes (*n* = 48) are sequentially added in the analysis. The number of genes (y-axis) is plotted against the number of genomes analyzed (x-axis) on a log–log scale. Represented as variation of their gene pool sizes upon sequential addition of the 48 *B. dentium* genomes.

In order to provide a comprehensive genomic overview of the species *B. dentium*, a pan-genome analysis was performed employing 48 *B. dentium* genomes: 38 of these represent complete and draft genomes retrieved from the National Center for Biotechnology Information (NCBI) (Sayers et al., 2022), while the remaining ten are *B. dentium* genomes herein sequenced. Pan-genome analysis was conducted using the pipeline Roary (Galaxy version 4.0.0rc1, Page et al., 2015), which determined a pan-genome consisting of 6,227 distinct gene families, of which 906 were shared among all assessed *B. dentium* genomes, thus constituting the core-genome of this species (Figure 1A; Table 2). According to the power regression function applied to the discovery of new gene families at each new genome addition, the *B. dentium* pan-genome shows an “open” trend with the number of novel gene families added for each additional *B. dentium* genome not reaching saturation (Figure 1B). Indeed, the power trend line do not reach a plateau (Figure 1B), which suggest an “open” pan-genome within the *B. dentium* species and indicates that its full genetic diversity has yet to be fully appreciated.

**Table 2:** Pan-genome analysis of 48 *B. dentium* representatives, consisting of the 10 strains used in this study as well as 38 additional, publicly available genomes.

Genomic classification	Number of gene families
Pan-genome	6,227
Core-genome	906
Soft-core-genome (≥94% strains)	1,520
Dispensable-genome	5,321

Due to the inclusion of draft genomes, our analysis returned a relatively low core-genome size when compared to other bifidobacterial species (Duranti et al., 2015; Duranti et al., 2016; Bottacini et al., 2018). To overcome the limitation of genome fragmentation introduced by draft sequences, we also computed a soft-core-genome estimated to represent gene families which occur in ≥94% of all available (i.e., 48) *B. dentium* representatives (Table 2), corresponding to 24.4% of the *B. dentium* pangenome, which is closer to the estimated core-genome of other bifidobacterial species (Duranti et al., 2015; Duranti et al., 2016; Bottacini et al., 2018). Notably, our comparative analysis also identified a total of 1,555 unique gene families (Supplementary Figure 2). Our pan-genome analysis furthermore revealed that mobile genetic elements, extracellular polysaccharide biosynthetic and carbohydrate utilization gene clusters, pili- and CRISPR-encoding genes, and genes encoding R/M systems constitute a significant portion of the accessory genome (i.e., gene functions not observed in all members of this species). A more detailed description of this mobilome and defensome follows in the next section.

### 3.3 *Bifidobacterium dentium* mobilome and defensome

The mobilome refers to the collection of genetic elements that can migrate within a genome and across different genomes (Siefert, 2009). Similar to other gut microbiota members, bifidobacteria harbor mobile genetic elements that may play a role in shaping their genomes providing rapid adaptation to environmental changes (Guglielmetti et al., 2013; Lugli et al., 2016). To examine the mobile element repertoire of *B. dentium*, the available 15 complete genome sequences were investigated for the presence of mobile DNA elements. This included genes encoding integrases, transposases, prophage sequences, integrated conjugative elements, and defense mechanisms against invading DNA or microbial competitors. These elements may play a role in the species’ adaptation to the oral and gut environment, thereby facilitating host colonization.

To identity DNA elements involved in movement of genetic material, e.g., transposases and integrases, hmmscan alignments against the PFAM database (http://ftp.ebi.ac.uk/pub/databases/Pfam; Mistry et al., 2021) were employed. Based on the obtained results, the number of identified transposase- and integrase-encoding genes ranged between 12 for *B. dentium* MB0076 and 30 for *B. dentium* MM0074 (Supplementary Table 3), which is a relatively low number compared to other bifidobacterial species, such as *B. breve* and *B. bifidum* (Bottacini et al., 2014; Duranti et al., 2015). The fully sequenced *B. dentium* chromosomes were also inspected for the presence of prophages using VirSorter2 (Guo et al., 2021) and this analysis identified 22 prophage sequences, ranging from zero to four across individual *B. dentium* chromosomes (salient features are listed in Supplementary Table 3). Looking at the intergenomic similarities between the identified prophage sequences using the VIRIDIC v1.1 tool (https://rhea.icbm.uni-oldenburg.de/viridic/; Moraru et al., 2020), two of these appeared to be unique (MB0148_ph1 and MB0076_ph2), while the remaining sequences were shown to cluster into seven similarity groups (Supplementary Figure 3). The screening of integrated conjugative elements (ICEs) using the ICE finder tool (https://bioinfo-mml.sjtu.edu.cn/ICEfinder/index.php), revealed the presence of 1 to 5 ICEs in the genomes of *B. dentium*, with an average number of 2 ICEs per genome (Supplementary Table 3).

With regards to the *B. dentium* defensome, CRISPR-Cas and Restriction Modification (R/M) systems were identified using CRISPRCasFinder (https://crisprcas.i2bc.paris-saclay.fr/CrisprCasFinder/Index) and the BLASTP (Altschul et al., 1990) alignment function of the REBASE database (https://rebase.neb.com/rebase/rebase.html; Roberts et al., 2023), respectively. Our analysis identified either one or two CRISPR-Cas systems encoded by each *B. dentium* genome (Supplementary Table 3). Screening of R/M systems among the 15 fully sequenced *B. dentium* strains revealed the presence of one to three R/M systems encoded by each genome with variability in terms of type (from Type I to IV) and genome location across the *B. dentium* strains (Supplementary Table 3). Exceptions to the latter are *B. dentium* strains MM0074, MB0372 and MB0385, which do not appear to encode any R/M systems, though they each specify at least one CRISPR-Cas system (Supplementary Table 3). While uncommon, the absence of R/M systems has been previously reported in bifidobacteria (Bottacini et al., 2018; Lugli et al., 2020a,b).

**Figure 2:**
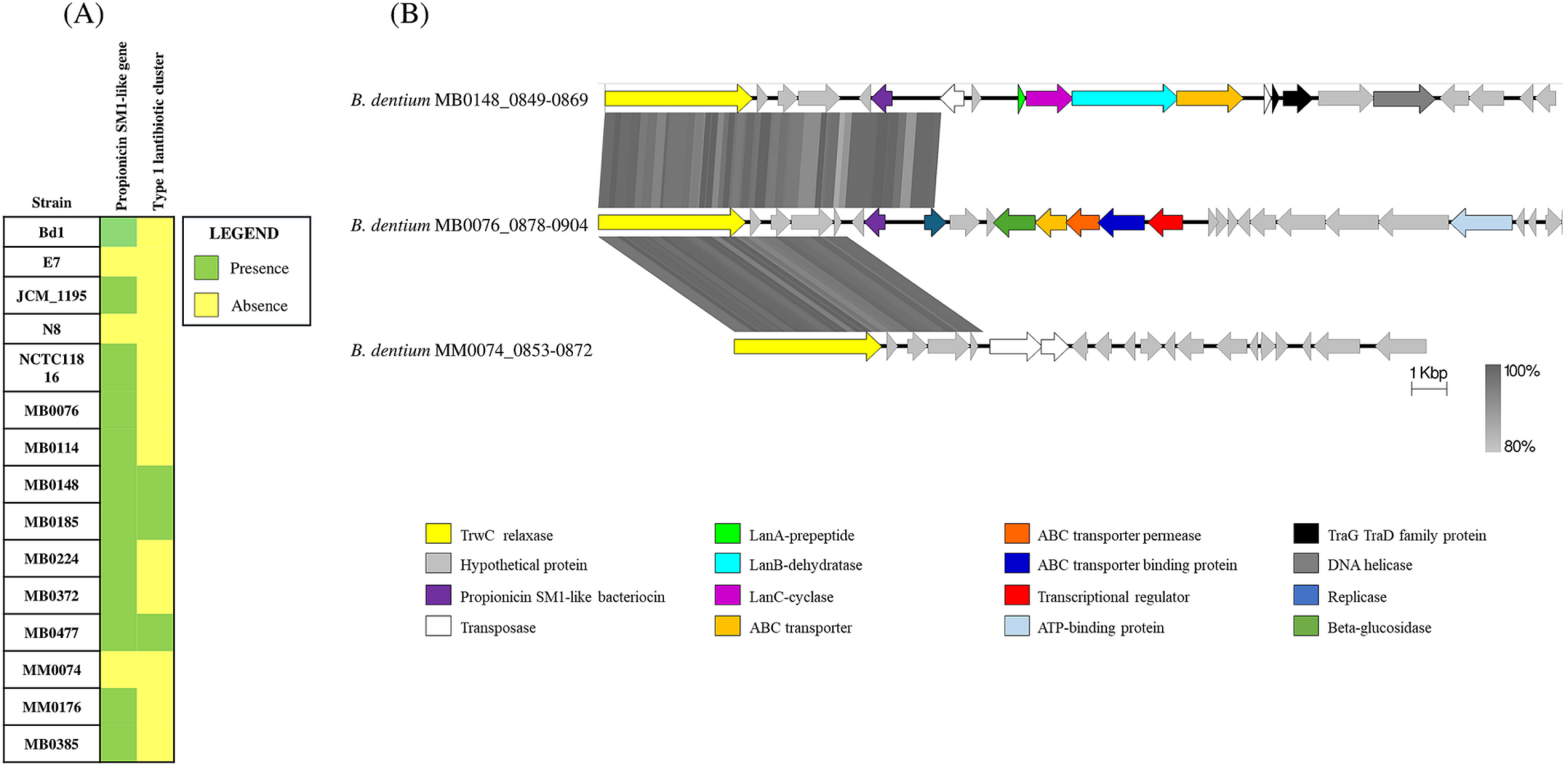
Bacteriocin biosynthetic gene clusters across 15 *B. dentium* strains. **(A)** Binary map representing the presence or absence of identified bacteriocin encoding clusters across the assessed *B. dentium* strains. **(B)** Linear comparison of the bacteriocin biosynthetic gene clusters across *B. dentium* strains. The comparison of gene clusters was created using Easyfig. The arrows represent coding sequences. Arrows pointing in the right direction are encoded on the forward strand, while arrows pointing in the left direction are encoded on the complementary strand. The size of the coding sequence is proportional to the length of the arrow. The predicted gene function is shown in the color legend. The percentage similarity between genes is indicated by the intensity of the gray connecting strand. The locus tag of the sequence is shown on the left-hand side.

A further investigation into the *B. dentium* defensome involved searching for genes responsible for bacteriocin biosynthesis in the 15 fully sequenced *B. dentium* genomes and performed using BAGEL4 (http://bagel4.molgenrug.nl/; van Heel et al., 2018; Sanchez-Gallardo et al., 2024a). The *in silico* analysis predicted the presence of a bacteriocin-encoding gene in the genomes of 12 out of the 15 assessed strains (Figure 2A). From a BLAST alignment (Camacho et al., 2009) the identified gene showed 35.71% identity (for 49% query cover) with a propionicin-SM1 gene from *Propionibacterium jensenii* (Miescher et al., 2000). The 12 *B. dentium* strains harbor the propionicin-SM1-like gene in the same locus within an IS-flanking and thus presumed mobile element, except for *B. dentium* strains MB0185 and MB0224 which contain this mobile element in a different chromosomal location. Further *in silico* analysis, using SignalP-5.0 (Bendtsen et al., 2004) and TMHMM-2.0 (Krogh et al., 2001), showed the presence of a signal peptide and a transmembrane helix in the propionicin-SM1-like gene sequence of the *B. dentium* strains, suggesting that this bacteriocin is secreted into the extracellular environment (Supplementary Figure 4).

In addition, BAGEL4 prediction (http://bagel4.molgenrug.nl/; van Heel et al., 2018) identified another bacteriocin biosynthesis-encoding gene cluster in *B. dentium* strains MB0477, MB0185 and MB0148 (Figure 2A). Functional assignment of the genes encoded in the cluster using BLASTP (Altschul et al., 1990) showed this bacteriocin gene cluster to represent a type 1 lantibiotic biosynthesis system composed of a prepeptide structural gene (encoded by *lanA*), two genes (*lanB* and *lanC*) that are predicted to encode enzymes for post-translational modifications of the bacteriocin prepeptide and one gene (*lanT*) responsible for transport. In *B. dentium* strains MB0477 and MB0148 the type 1 lantibiotic cluster is located within the same predicted mobile element as that containing the propionicin-SM1-like gene (Figure 2B). The different position of the cluster in *B. dentium* MB0185 is probably due to the presence of two transposases that flank the type 1 lantibiotic gene cluster, enabling its mobilization (Figure 2B). In this case, no signal peptide or transmembrane helix was found, indicating that this peptide is probably modified, processed and secreted from the cell by the ABC transporter. The genes that encode these bacteriocins display a lower G + C content (45%) when compared to the average G + C content (58.5%), supporting the notion of Horizontal Gene Transfer (HGT) acquisition.

### 3.4 Comparative genomics of extracellular polysaccharide-biosynthesis related gene clusters

In order to further investigate *B. dentium*-host interaction, we identified the diversity and distribution of exo- or cell wall-polysaccharide (collectively abbreviated here as EPS) specifying loci in *B. dentium*. Indeed, EPS has been shown to be critical in host–microbe interactions by contributing to the bacterium’s immunomodulatory activity (Hickey et al., 2021; Fanning et al., 2012), enhancing adherence to the host intestinal mucosa (Inturri et al., 2017) and providing protection under stressful conditions (Kelly et al., 2020).

**Figure 3:**
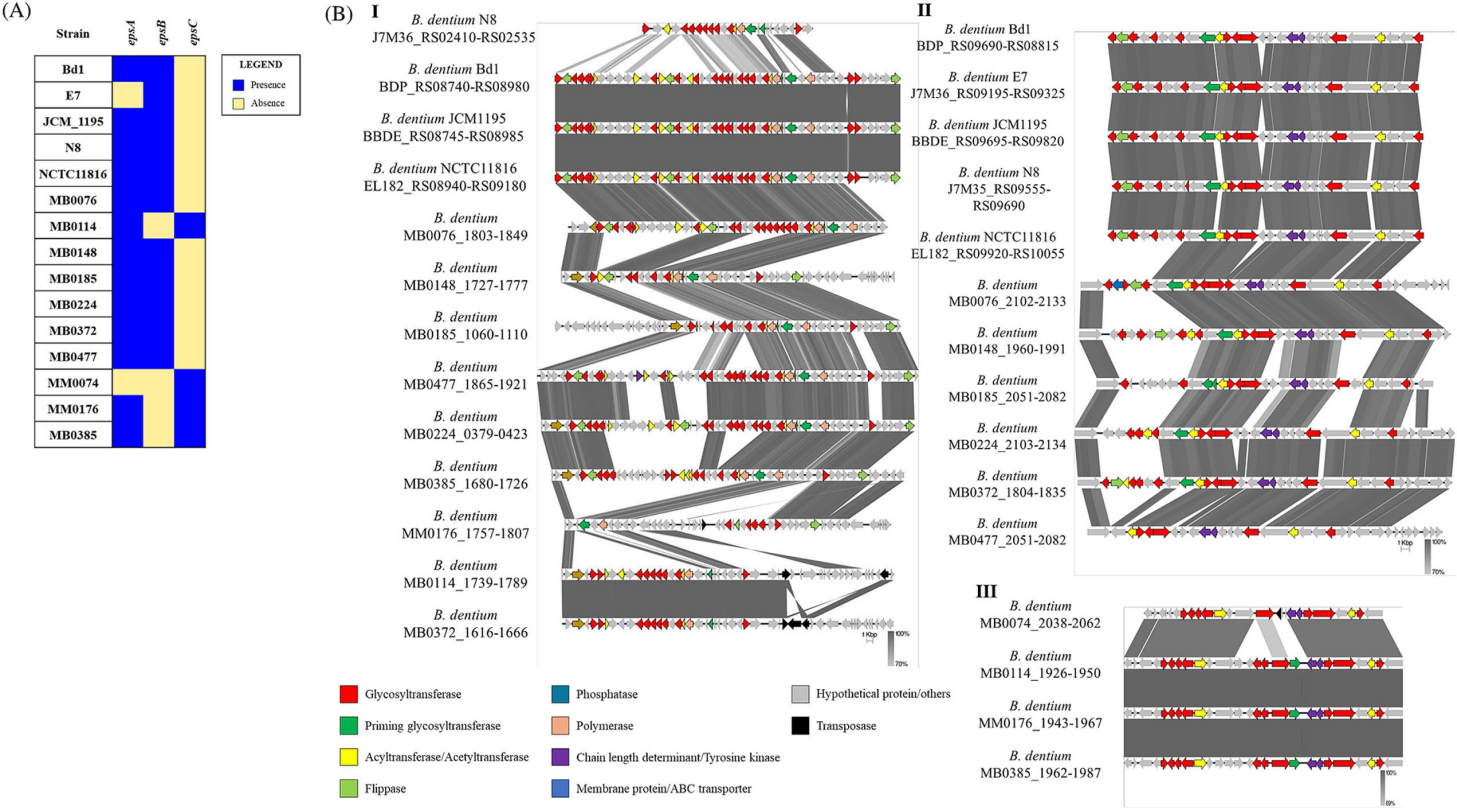
Predicted exopolysaccharide (EPS)-biosynthesis loci identified in the genomes of 15 *B. dentium* strains. **(A)** Binary map representing the presence or absence of the EPS loci identified across the assessed *B. dentium* strains. **(B)** Linear comparison of EPS loci *epsA* (panel **I**), *epsB* (panel **II**) and *epsC* (panel **III**) across *Bifidobacterium dentium* strains as created using Easyfig. Arrows represent coding sequences. Except in the case of the *epsA* loci of *B. dentium* MB0185 and *B. dentium* MB0224, which were reversed to aid visualization, arrows pointing in the right direction are encoded on the forward strand, while arrows pointing in the left direction are encoded on the complementary strand. The size of the coding sequence is proportional to the length of the arrow. The predicted gene function is shown in the color legend. The percentage similarity between genes is indicated by the intensity of the gray connecting strand. The locus tag of the sequence is shown on the left-hand side.

The presence of possible EPS clusters was verified *in silico* in the 15 fully sequenced *B. dentium* strains using hmmscan alignments against the PFAM database (http://ftp.ebi.ac.uk/pub/databases/Pfam; Mistry et al., 2021). The search for genomic regions encoding genetic elements that define an EPS cluster (Bottacini et al., 2018), such as several glycosyltransferases, a flippase or an ABC transporter, a priming glycosyltransferase, and a chain-length regulator resulted in the identification of three different EPS-biosynthesis-related loci across the assessed *B. dentium* genomes, which were named *epsA*, *epsB* and *epsC* according to the chromosomal position and cluster size (Figure 3). The analysis resulted in the identification of two EPS loci in 13 out of 15 *B. dentium* strains, with *epsA* being conserved in these 13 *B. dentium* genomes. Strains MM0074 and E7 harbor a single EPS locus (represented by either *epsB* or *epsC*, respectively). The *epsC* locus was also present in the genome of *B. dentium* strains MB0114, MB0176, and MB0385 in conjunction with *epsA*. The other *B. dentium* genomes were shown to contain *epsB* along with *epsA* (Figure 3A).

Gene content comparison of representatives for each of these three *eps* loci revealed high variability for *epsA* across the *B. dentium* strains in terms of both gene content and locus size, ranging between 30 to 60 Kb (Figure 3BI), while *epsB* and *epsC* (being approximately 20 and 30 Kb in size, respectively) exhibit less genetic diversity (Figures 3BII–III). Notably, the chromosomal position of these three loci across the strains is mostly conserved, with *epsA* always flanked by *epsB* or *epsC*. Only *B. dentium* MB0185 and MB0224 show a different chromosomal position of the EPS loci due to a chromosomal rearrangement in their genome (Supplementary Figure 1A).

### 3.5 Potential for pilus formation in *Bifidobacterium dentium*

Evaluation of host colonization factors in *B. dentium* was performed by searching for gene clusters responsible for pilus biosynthesis, which are surface appendages involved in interaction between gut microbes and the intestinal mucosa (Foroni et al., 2011; O’Connell Motherway et al., 2011). For this purpose, the complete genomes of 15 *B. dentium* strains were searched for the presence of possible pilus biosynthesis-encoding clusters using hmmscan alignments against the PFAM database (http://ftp.ebi.ac.uk/pub/databases/Pfam; Mistry et al., 2021). In accordance with literature, *B. dentium* harbors multiple sortase-dependent pilus clusters (Supplementary Table 4) and a type IVb tight adherence (Tad) pilus-encoding cluster, similar to the one conserved in *B. breve* (O’Connell Motherway et al., 2011). Expression of sortase-dependent pilus biosynthesis-encoding genes in the genus *Bifidobacterium* may be influenced by DNA polymerase-mediated slippage, promoted by the presence of a long G-tract sequence upstream the first gene of the cluster (Penno et al., 2022). *In silico* analysis of the ten *B. dentium* genomes indeed revealed a number of clusters associated with such poly-G stretches (Supplementary Table 4), which generates diversity in the expression of pili structures that may constitute a target for the host’s immune system (Phillips et al., 2019; Penno et al., 2022).

The greater number of pilus-encoding gene clusters in the assessed strains (ranging from five to eight across individual *B. dentium* chromosomes, Supplementary Table 4) when compared to other bifidobacterial species (Foroni et al., 2011; Penno et al., 2022), corroborates previous findings that *B. dentium* possess a higher number of adhesion abilities compared to other bifidobacteria which may provide ways to adapt to different ecological environments (i.e., oral cavity and intestine) (Foroni et al., 2011).

### 3.6 Glycoside hydrolase identification and carbohydrate utilization in *Bifidobacterium dentium*

#### 3.6.1 Glycoside hydrolase identification

**Figure 4:**
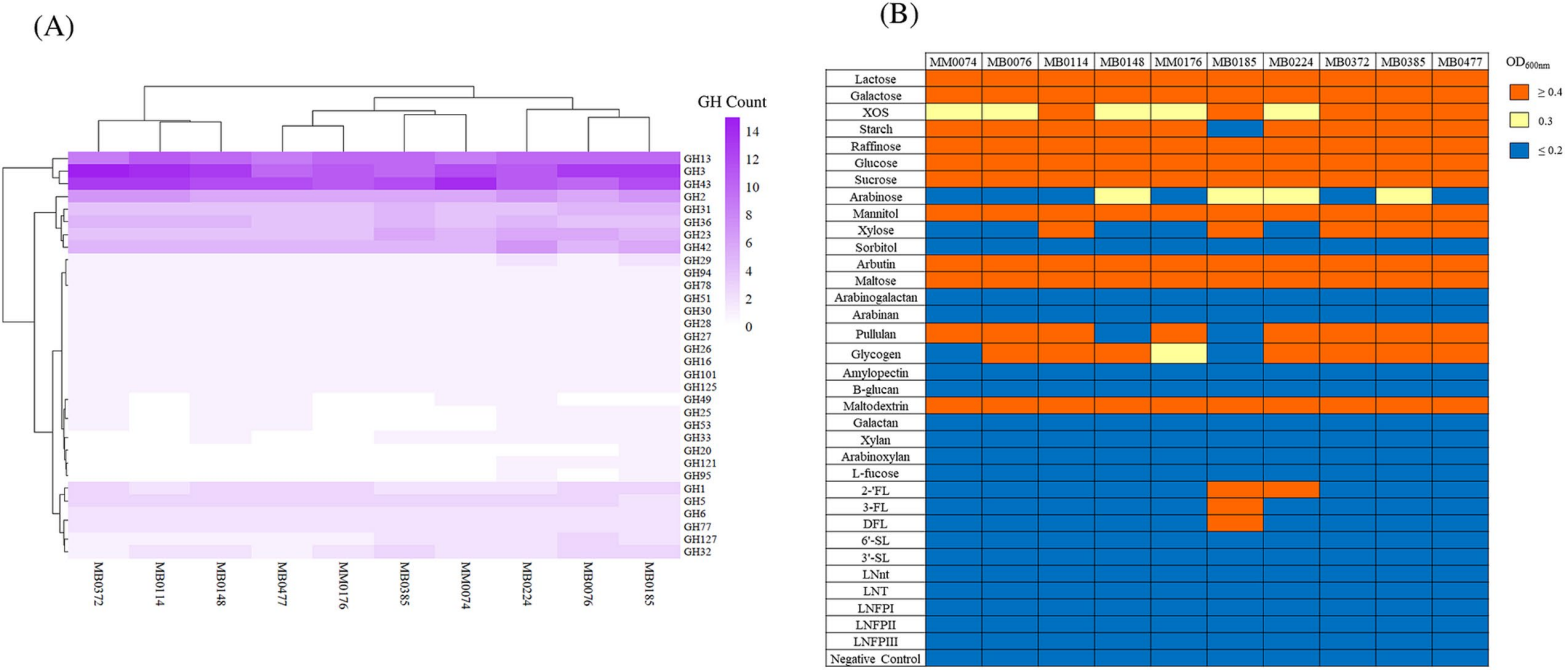
**(A)** *B. dentium* glycosyl hydrolase (GH) profile. Heatmap representing the distribution of glycosyl hydrolases (GHs) among 10 *B. dentium* strains. Hierarchical clustering based on the identified GH families is reported. **(B)** *B. dentium* carbohydrate fermentation profiles on 34 glycans, as the sole carbon source. The cut-off for good growth was OD600nm ≥ 0.4, while OD_600nm_ values equal to 0.3 was designated as intermediate growth and below 0.2 classified as no growth. The negative control demonstrates a lack of growth in the absence of carbohydrate addition.

In order to explore carbohydrate fermentation abilities of *B. dentium*, glycosyl hydrolase (GH) identification and growth experiments were performed focusing on the ten newly sequenced *B. dentium* strains (as we had only access to these strains for such growth analyses). GHs were identified through *in silico* prediction using dbCAN (Zhang et al., 2018) for these ten strains. This prediction revealed that the number of GH-encoding genes across the strains ranged from 79 in *B. dentium* MM0176 to 94 in *B. dentium* MB0185, representing 32 distinct GH families, the prevalence and abundance of which is depicted in Figure 4A. *B. dentium* harbors a high number of genes predicted to encode GHs belonging to the GH3, GH13 and GH43 families (average of 12 genes per genome whose encoded products belong to the GH3 and GH43 families, and 10 genes encoding GHs belonging to the GH13 family) (Figure 4A), which are generally involved in the degradation of plant-derived glycans, such as xylose (Xia et al., 2015), xylan (Dotsenko et al., 2012), arabinoxylan-oligosaccharides (Saito et al., 2020), starch, amylose or arabinogalactan (Milani et al., 2015). Of note, multiple members belonging to GH2 family coding for β-galactosidases or β-mannosidases were present in the assessed strains (corresponding to an average of 6 genes per genome) (Figure 4A).

#### 3.6.2 Plant-derived glycan utilization

Following the identification of glycoside hydrolase-encoding genes, growth profiles were used to establish associations between possible gene(s) or gene cluster(s) responsible for the utilization of both simple and complex plant-derived carbohydrates (lactose being the exception, which was used as a positive control) in *B. dentium*. In order to assess *B. dentium* growth abilities, growth assays were performed by performing OD_600nm_ measurements following 24 h of anaerobic growth at 37°C on 34 different carbohydrates, each being used as the sole carbon source. The cut-off point for good growth was designated to be an OD_600nm_ value equal to or larger than 0.4, while OD_600nm_ values between 0.2 and 0.4 as intermediate growth and below 0.2 classified as poor/no growth (Bottacini et al., 2018).

As shown in Figure 4B, consistent growth was observed for all 10 strains on lactose, galactose, glucose, sucrose, raffinose, and maltose, which is in accordance with what has been observed in other human-derived bifidobacterial species (Duranti et al., 2015; Duranti et al., 2016; Arboleya et al., 2018). None of the tested *B. dentium* strains was shown to grow on the plant-derived polysaccharides arabinoxylan, arabinogalactan, arabinan, xylan and galactan (Figure 4B). In contrast, most of the analyzed strains displayed growth on starch and pullulan (Figure 4B). Variable growth was furthermore observed across the examined strains on xylo-oligosaccharide (XOS), xylose, glycogen and arabinose (Figure 4B). Moreover, *B. dentium* was shown to able to grow on mannitol only, and failed to elicit growth on sorbitol (Figure 4B).

Subsequently, in order to match the above mentioned carbohydrate-based growth phenotypes with genotype, we performed comparative genome-based and HPAEC-PAD analyses, which were further complemented and validated by literature-derived information.

As expected, gene clusters involved in raffinose and sucrose utilization were identified in all assessed strains, in both cases exhibiting high sequence similarity and common gene order to raffinose and sucrose utilization gene clusters previously described in bifidobacteria (Supplementary Figures 5A,B; O’Connell et al., 2013; O’Connell et al., 2014; Tanno et al., 2019; Ryan et al., 2005). Like the *B. breve* UCC2003 *raf* cluster, *B. dentium* encodes an ABC-type transport system presumed to be responsible for intracellular transport of raffinose, which is then hydrolysed by a GH36 family α-galactosidase into sucrose and galactose. The α-galactosidase-encoding gene is probably regulated by a ROK-type regulator encoded by an adjacent gene (Supplementary Figure 5A; O’Connell et al., 2014). Sucrose utilization in *B. dentium* seems to be linked to a gene cluster which displays high similarity to a cluster involved in sucrose and FOS utilization in *B. breve* and *B. longum* (Supplementary Figure 5B; Ryan et al., 2005; Tanno et al., 2019), also suggesting a possible utilization of FOS by *B. dentium*. The β-fructofuranosidase encoded in the cluster (Supplementary Figure 5B) is predicted to be involved in the intracellular hydrolysis of the β(2-1) glycosidic linkage of sucrose and FOS, releasing glucose and fructose. A flanking permease-encoding gene is presumed to be responsible for sucrose (or FOS) internalization. Upstream of the gene encoding the sugar transporter, an ORF encoding a probable LacI-type transcriptional regulator was identified, most likely involved in the sugar-mediated transcriptional control of this cluster (Supplementary Figure 5B).

In relation to growth on starch and pullulan sugars (Figure 4B), a gene encoding a predicted type II pullulanase similar to that characterized for *B. adolescentis* P2P3 (Kim et al., 2021) and *B. breve* UCC2003 (O’Connell Motherway et al., 2008) was present in all assessed *B. dentium* strains. SignalP v5.0 analysis (Bendtsen et al., 2004; https://services.healthtech.dtu.dk/services/SignalP-5.0/) identified a signal peptide sequence in this putative pullulanase, suggesting an extracellular activity of this glycosyl hydrolase. As described by Kim and colleagues, the pullulanase harbors (i) an α-amylase domain at its N-terminus that can act on α-1,4-glucosidic bonds in polysaccharides such as starch, and (ii) a pullulanase type I domain at its C-terminus involved in the hydrolysis of α-1,6-glucosidic bonds as in pullulan, with three carbohydrate-binding modules located between these two domains. The apparent inability of *B. dentium* MB0185 to grow on either starch or pullulan, and *B. dentium* MB0148’s failure to grow on pullulan (Figure 4B), may be related to genetic variations that influenced the expression or activity of the enzyme or one of its domains.

A putative XOS and xylose utilization cluster was found in all assessed *B. dentium* strains, harboring genes encoding enzymes previously found to be involved in xylo-oligosaccharide utilization in *B. dentium* (Supplementary Figure 5C; Lee et al., 2022). The cluster harbors several transporter-encoding genes that are predicted to be involved in sugar internalization, as well as other enzymes essential for XOS degradation into its monomeric constituent xylose (Lee et al., 2022). Xylose is converted into xylulose by a xylose isomerase, and phosphorylated by xylulose kinase to xylulose 5-phosphate, which then enters the bifid shunt pathway (Gilad et al., 2010). Since dissimilar growth was observed in XOS (Figure 4B), an analysis of XOS internalization was performed on a faster (*B. dentium* MB0114) and slower (*B. dentium* MM0074) grower strain using HPAEC-PAD analysis. The supernatant composition of *B. dentium* MB0114 and MM0074 were analyzed after 24 h of growth on 1% XOS, and, as Supplementary Figure 6 reports, there is no apparent difference in XOS concentration between the two supernatants, suggesting that the difference in growth between the two strains is not related to XOS transport.

All strains harbor a gene cluster similar to a gene cluster previously shown to be associated with mannitol utilization by *B. dentium* Bd1 (Ventura et al., 2009). This putative *B. dentium* mannitol cluster harbors a transporter belonging to the major facilitator superfamily (MFS), possibly involved in mannitol transport, and alcohol dehydrogenase-encoding genes, involved in mannitol metabolism. Indeed, mannitol is converted into fructose by an alcohol dehydrogenase, and then phosphorylated by fructokinase to fructose-6-phosphate, which is then further processed by the bifid shunt pathway (Caescu et al., 2004; Ortiz et al., 2017; Wisselink et al., 2002).

#### 3.6.3 Human milk oligosaccharide utilization

We also assessed the ability of our *B. dentium* strains to grow on human milk oligosaccharides (HMOs) (2’-FL, 3-FL, DFL, LNFPI, LNFPII, LNFPIII, LNT, and LnNT) through growth assays performed as described in the previous section. Notably, our findings revealed that *B. dentium* strains MB0185 and MB0224 exhibit growth on some of the fucosylated HMOs (Figure 4B), while no appreciable growth was observed for the other strains or other HMOs (Figure 4B; Supplementary Figure 7). In order to provide a more detailed growth analysis, growth curves on a particular HMO as the sole energy source, were performed (Supplementary Figure 7). Growth curves showed that both *B. dentium* strain MB0185 and MB0224 grow more or less equally well on 2′-fucosyllactose (2’-FL) (Supplementary Figure 7B; OD_620nm_ of 0.6 and 0.5, respectively, after 45 h). However, when *B. dentium* MB0224 is grown on 3-fucosyllactose (3-FL), it exhibits a lower growth rate compared to *B. dentium* MB0185, which reached OD_620nm_ of 0.4 after 19 h of growth, whereas *B. dentium* MB0224 reached the same OD_620nm_ after 28 h of cultivation (Supplementary Figure 7C). When examining growth on difucosyllactose (DFL), just *B. dentium* MB0185 was demonstrated to exhibit appreciable growth on this HMO (OD_620nm_ of 0.4 after 45 h), while *B. dentium* MB0224 was shown to be unable to grow on this HMO (OD_620nm_ of 0.1 after 45 h) (Supplementary Figure 7D).

### 3.7 Comparison of fucosyllactose utilization genes across the *Bifidobacterium* genus

**Figure 5:**
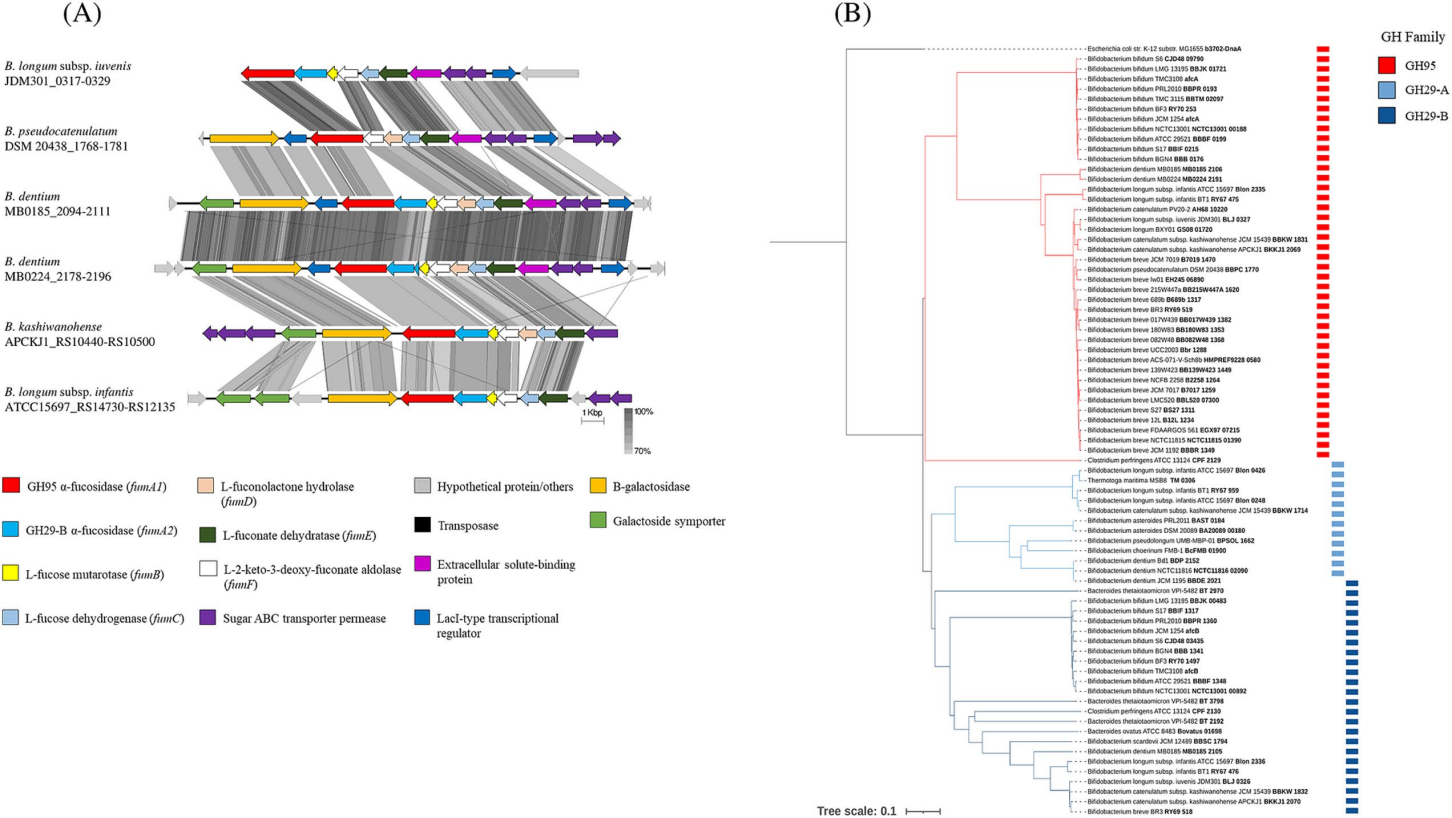
**(A)** Linear comparison of fucosyllactose metabolism genes across bifidobacterial strains. The arrows represent coding sequences. Except in the case of *B. longum* subsp*. iuvenis* JDM301, *B. dentium* MB0185 and *B. dentium* MB0224, which were reversed to aid visualization, arrows pointing in the right direction are encoded on the forward strand, while arrows pointing in the left direction are encoded on the complementary strand. The size of the coding sequence is proportional to the length of the arrow and the predicted gene function is shown in the color legend. The percentage similarity between genes is indicated by the intensity of the gray connecting strand. The locus tag of the locus is shown on the right-hand side. **(B)** Phylogenetic relationships of GH95 and GH29 α-fucosidases. The phylogenetic tree was built using the neighbor-joining method using CLUSTAL_W and visualized through iTOL. The clade of the GH95 family is highlighted in red, and the clade of the GH29 family is highlighted in blue and subdivided into subclades GH29-A (light blue) and GH29-B (dark blue).

Growth assays revealed that two *B. dentium* strains (*B. dentium* MB0185 and MB0224) capable of utilizing 2’-FL and/or 3-FL/DFL as sole carbon sources. This finding unveils a previously unrecognized metabolic trait in *B. dentium*, expanding our understanding of its adaptability and functional potential. Integration of genomic analysis, particularly GH prediction, uncovered that these two *B. dentium* strains possess a gene cluster, which is highly homologous to fucose/fucosyllactose utilization clusters identified in several infant-derived bifidobacterial species (Figure 5A; James et al., 2019; Sanchez-Gallardo et al., 2024b; Sela et al., 2012; Arboleya et al., 2018). This cluster was absent in other assessed *B. dentium* strains, highlighting its unique presence in *B. dentium* MB0185 and MB0224.

Genome analysis of *B. dentium* MB0185 and MB0224 revealed the presence of several transposase/integrase-encoding genes flanking their predicted fucosyllactose-utilization gene clusters, which suggests that this region represents a mobile genetic element (Supplementary Figure 8), and which could contribute to its transfer and *B. dentium* adaptability in different environments. The *B. dentium* fucosyllactose-utilization cluster appears to encode all enzymes required for fucosyllactose (and its constituents lactose and fucose) metabolism as outlined below (Figure 5A; Supplementary Figure 9), highlighting its potential to process HMOs in the gut. The first step of the proposed fucosyllactose metabolic pathway involves the internalization of the fucosylated HMOs through an ABC-type transporter encoded within the cluster and represented by genes specifying two sugar permeases and an extracellular solute-binding protein. Two specialized uptake systems for the internalization of HMOs, with distinct functionality, were previously described in bifidobacterial species, as well as *B. dentium* (Ojima et al., 2022; Sakanaka et al., 2019). The two transporters possess identical permease subunits but, due to their divergent solute-binding protein domains, their ligands specificity differs, with the type 1 being able to transport only 2’-FL and 3-FL, while the type two imports a wider variety of HMOs (2’-FL, 3-FL, LNFPI and lactodifucotetraose; Sakanaka et al., 2019). Even though a homolog of the type 2 transporter was identified in *B. dentium* strains (Ojima et al., 2022), the *B. dentium* strains used in our study showed a lack of growth on LNFPI and LNFPII (Figure 4B) suggesting that *B. dentium* MB0185 and MB0224 possess a different ABC transport system for fucosylated HMOs (possibly a representative of the type 1 identified in *B. infantis* and *B. breve* strains; Ojima et al., 2022).

The fucosylated HMOs are then believed to be hydrolyzed by two α-fucosidases (FumA1 and FumA2) releasing fucose and lactose, which in turn is further metabolized by a β-galactosidase encoded in the cluster and the reaction products (i.e., galactose and glucose) are processed by the bifid shunt pathway. Several enzymes encoded by the cluster (FumB, FumC, FumD, FumE and FumF) then process the released L-fucose into L-lactaldehyde and pyruvate, which are further metabolized releasing lactate, acetate, formate and L-1,2-propanediol (corresponding genes and activity of the above mentioned enzymes are indicated in Supplementary Figure 9; James et al., 2019). This metabolic pathway is probably regulated by a LacI-type transcriptional regulator also encoded by the *B. dentium* fucosyllactose-utilization cluster (Figure 5A).

A phylogenetic analysis of the *B. dentium* FumA1 and FumA2 proteins confirmed that these α-fucosidases belong to two different GH families: GH95 and GH29-B (Figure 5B), with distinct hydrolytic activity.

The limited ability and inability of *B. dentium* MB0224 to grow on 3-FL and DFL, respectively (Supplementary Figures 7C,D) is explained by the presence of several mutations in the *fumA2* gene, which harbors a frameshift and various nucleotide substitutions across the gene, when compared to the *B. dentium* MB0185 *fumA2*.

In order to investigate the enzymatic activity of the GH95 (FumA1) and GH29B (FumA2) α-fucosidases encoded by *B. dentium* MB0185 toward HMOs, the predicted fucosidase-encoding genes, *fumA1_bd185_* and *fumA2_bd185_*, were cloned into *E. coli* BL21 using plasmid pET28b and the proteins were overproduced and purified as His-tagged versions. FumA1_bd185_ and FumA2_bd185_ were then incubated with various potential substrates, 2’-FL, 3-FL, DFL, LNFPI, LNFPII or LNFPIII, after which reaction products were analyzed by HPAEC-PAD (see Methods section).

**Figure 6:**
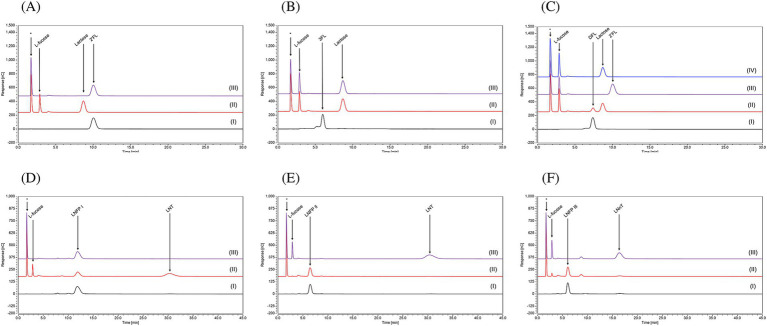
Chromatogram profiles using High-Performance Anion Exchange Chromatography with Pulsed Amperometric Detection (HPAEC-PAD) of **(A)** 2’-FL, **(B)** 3-FL, **(C)** DFL, **(D)** LNFPI, **(E)** LNFPII and **(F)** LNFPIII in (I) Milli-Q water; or in MOPS buffer with (II) FumA1_bd185_, (III) FumA2_bd185_, (IV) FumA1_bd185_ and FumA2_bd185_ together. This technique was used to separate the HMOs based on their charge under alkaline conditions and then detect them measuring the electric current produced when the carbohydrates are oxidized at the detector surface, allowing highly sensitive identification. (*) indicates a peak which is due to the MOPS buffer used for the enzymatic assays.

Purified FumA1_bd185_ was shown to remove the L-fucose moiety of the substrates 2’-FL, 3-FL and DFL (Figures 6A–C), demonstrating hydrolytic activity toward α-1,2- and α-1,3-linkages (see Supplementary Figure 10). However, when FumA1_bd185_ was incubated with DFL, incomplete cleavage of this HMO was observed, suggesting a slower hydrolytic activity when both the α-1,2- and α-1,3-linkages are present (Figure 6C). When incubated with larger substrates, such as LNFPI, LNFPII, or LNFPIII, purified FumA1_bd185_ elicited an apparently slow hydrolytic activity toward α-1,2-linkages, as the chromatogram profile of LNFPI displays an incomplete cleavage of the HMO into L-fucose and LNT (Figure 6D), while no breakdown of LNFPII and LNFPIII was observed (Figures 6E,F). The lower or absent hydrolytic activity of FumA1_bd185_ with larger substrates (i.e., LNFPI and LNFPIII) may result from the larger substrate size that affects the enzyme-substrate interactions. Indeed, AlphaFold predicted structures show that FumA1_bd185_ possesses a binding site cavity that may not accommodate larger substrates (Supplementary Figure 11A). The predicted local distance difference test (pLDDT) scores of the AlphaFold models and the predicted aligned error (PAE) maps are shown in Supplementary Figure 12.

In contrast, purified FumA2_bd185_ was shown to elicit selective hydrolytic activity toward α-1,3-linkages since no substrate degradation was observed when this enzyme was incubated with 2’-FL (Figure 6A), whereas release of L-fucose was observed when incubated with 3-FL (Figure 6B). Moreover, this α-1,3-selectivity was observed also when incubated alone with DFL, as L-fucose and 2’-FL were the reaction products (Figure 6C). The expected complementary hydrolytic activity of FumA1_bd185_ and FumA2_bd185_ was confirmed by the HPAEC chromatogram profile of DFL when both α-fucosidases were incubated with this HMO, indeed complete removal of the lactose moiety was observed (Figure 6C). Following FumA2_bd185_ incubation with LNFPI no degradation of the oligosaccharide structure was observed (Figure 6D). However, when FumA2_bd185_ was incubated with LNFPII or LNFPIII complete removal of the L-fucose moiety was observed (Figures 6E,F), demonstrating hydrolytic activity toward α-1,3- and also α-1,4-linkages (Supplementary Figure 10).

## 4 Discussion

Several studies have explored the ecological niche that *B. dentium* inhabits, based on the presumption that this microorganism naturally inhabits the human oral cavity (Manome et al., 2019; Ventura et al., 2009; Kaur et al., 2013). However, *B. dentium* presence in human fecal samples across different age groups suggests its natural habitat extends beyond the oral cavity, though its impact in the gut remains unclear (Duranti et al., 2017; Kato et al., 2017). In the present study, we explored the newly obtained genetic features of ten *B. dentium* isolates combined with 38 publicly available *B. dentium* genomes. Comparative genome analysis was performed in order to assess host-interaction features and carbohydrate metabolic abilities, giving the currently most comprehensive genomic analysis of this species. Our analysis reveals that members of the *B. dentium* taxon possess a larger chromosome (at an average of 2.6 Mbp) compared to other human gut bifidobacterial species, such as *Bifidobacterium breve, Bifidobacterium bifidum*, *Bifidobacterium adolescentis*, and *Bifidobacterium longum* subsp. *longum* (Bottacini et al., 2014; Duranti et al., 2015; Duranti et al., 2016; Arboleya et al., 2018). This increased genome size appears to be driven by an expanded accessory genome, which may be connected to *B. dentium*’s ability to colonize multiple niches (i.e., oral cavity and gut).

The prediction of an “open” pan-genome and the high number of unique genes identified in this taxon suggest a high genome variability compared to other bifidobacterial species (Bottacini et al., 2014; Duranti et al., 2015; Duranti et al., 2016; Arboleya et al., 2018). Our analysis also identified numerous mobile elements, such as genes specifying integrases and transposases, integrative and conjugative elements, prophages sequences and defense mechanisms against mobile DNA acquisition (CRISPR-Cas and R/M systems), highlighting HGT as a key feature of *B. dentium*’s evolution. These findings, along with the identification of structural chromosomal rearrangements in the genome of *B. dentium* MB0224 and MB0185, which may be caused by recombination between *IS* elements, indicate that (some) *B. dentium* strains possess a structurally less stable genome compared to other bifidobacteria.

The genetic diversity in the *B. dentium* taxon is also evident from the analysis of gene clusters encoding functions related to host interaction (e.g., bacteriocin biosynthetic genes, pilus-encoding gene clusters, and extracellular polysaccharide encoding clusters). *In silico* screening identified three EPS-biosynthesis-related loci (*epsA*, *epsB*, and *epsC*), consistent with what has previously been observed in various Gram-positive bacteria (Bottacini et al., 2018; Fanning et al., 2012; Dan et al., 2009; Guérin et al., 2022). The genetic diversity and structural variations in these EPS clusters suggest a potential role in strain-specific adaptations to different host environments. Furthermore, most of the assessed *B. dentium* strains have acquired a variety of bacteriocins, which may influence microbial competition and host colonization. Most strains harbor a propionic-SM1-like bacteriocin gene, and some also possess a type 1 lantibiotic cluster, which appears to have been acquired by HGT. Moreover, *B. dentium* harbors multiple sortase-dependent pilus clusters, some associated with poly-G stretches, suggesting a role in adhesion to host tissues and potentially influencing colonization dynamics within the oral cavity and gut (Penno et al., 2022).

The pan-genome of *B. dentium* harbors a large repertoire of GH families predicted to be involved in the metabolism of a variety of glycans, which supports the notion that *B. dentium* is able to colonize multiple niches. Growth experiments, in combination with a comparative genome analysis, confirm *B. dentium*’s ability to metabolize various mono- and oligo-saccharides, consistent with previous findings (Lugli et al., 2020a; Engevik et al., 2021a). Even though multiple members of the GH3 and GH43 families are encoded by all strains, suggestive of an ability to degrade complex plant glycans no growth was observed on any of these polysaccharides. *In silico* analysis highlighted these GH3 and GH43 members lack a secretion signal and are thus presumed to represent cytoplasmic enzymes. As observed in other bifidobacterial species (Yokoi et al., 2022; Munoz et al., 2022), it is possible that *B. dentium* indirectly utilizes these complex plant-derived glycans through cross-feeding interactions, where these glycans are initially degraded to oligosaccharides by other members of the intestinal microbial community by means of extracellular hydrolytic activities. Such released oligosaccharides may then internalized and further utilized by *B. dentium* through cytoplasmatic GHs (i.e., GH3 and GH43). The conservation of GH families associated with the degradation of complex plant glycans suggests that *B. dentium* is adapted to niches where plant glycan breakdown products are abundant, taking advantage of the metabolic output of the surrounding microbial community to support its own colonization and persistence.

Of note, two *B. dentium* genomes (*B. dentium* MB0185 and MB0224) encompass a gene cluster which is involved in the metabolism of fucosylated human milk oligosaccharides (HMOs), and which is similar to those present in genomes of other bifidobacterial species/strains (James et al., 2019; Sanchez-Gallardo et al., 2024b). This metabolic capability, previously unrecognized in *B. dentium*, suggests an adaptation mechanism that may expand its ecological niche. The absence of this gene cluster in other *B. dentium* strains suggests that HMO utilization is not a conserved trait within the species but rather a strain-specific adaptation. This may explain why *B. dentium* is not prevalent in infants compared to certain other bifidobacterial (sub)species (Feehily et al., 2023). As shown in Figure 5A, fucosylated HMO utilization gene clusters differ among various *Bifidobacterium* species. In *B. dentium* the fucosyllactose utilization gene cluster is similar to that of *B. kashiwanohense* (James et al., 2019), featuring two glycosyl hydrolases (GH29 and GH95) required for 2’-FL, 3-FL metabolism (Garrido et al., 2016; James et al., 2019; Shani et al., 2022), and, as demonstrated in this study, are also required for DFL utilization.

These findings open several important avenues for future research. Indeed, understanding the genetic mechanisms underlying *B. dentium*’s niche adaptation and its ability to utilize a diverse range of carbohydrates, including HMOs, will be crucial to explore its role in both the oral and gut microbiomes. Moreover, the high level of genetic variability and horizontal gene transfer in *B. dentium* suggests that its metabolic traits and ability to interact with the host may evolve rapidly, which could have implications for its ecological and functional roles in the microbiota.

## 5 Conclusion

Through comparative genomic analysis, the genomic diversity of *B. dentium* was elucidated revealing an open pan-genome and a high level of genetic diversity linked to HGT events. The identification of a large chromosome (2.6 Mbp) compared to other bifidobacterial species along with the presence of numerous GH families and a complex range of host-colonization factors indicate that this species is adapted to colonize multiple niches and to compete with other colonizers. GH screening identified a large glycobiome with an apparent focus on the degradation of plant polysaccharides, with GH3, GH13, and GH43 being the predominant families observed. However, *B. dentium* failed to grow on complex plant polysaccharides, which, as already observed in other bifidobacteria (Yokoi et al., 2022; Munoz et al., 2022), suggests utilization of (plant-derived) complex glycans via resource-sharing and cross-feeding interactions. Moreover, most of the tested *B. dentium* strains failed to metabolize HMOs except for *B. dentium* MB0185 and *B. dentium* MB0224. The exploration of a fucosyllactose utilization locus encoded within an apparent mobile element in *B. dentium* MB0185 and MB0224 identified them as the first *B. dentium* strains capable of this activity.

## Data Availability

All sequences generated in this study have been submitted to the NCBI database under BioProject accession number PRJNA1073879.
