# Supplementary material for: Gene-trait matching among Bifidobacterium dentium strains reveals various glycan metabolism loci including a strain-specific fucosyllactose utilization cluster
Source: Front Microbiol. 2025 May 12;16:1584694. doi: 10.3389/fmicb.2025.1584694 (PMC12104195; doi:10.3389/fmicb.2025.1584694)
Supplement: Supplementary file 1 [file Data_Sheet_1.docx]

Supplementary Material

# Supplementary Figures and Tables

## Supplementary Figures

(A)

**
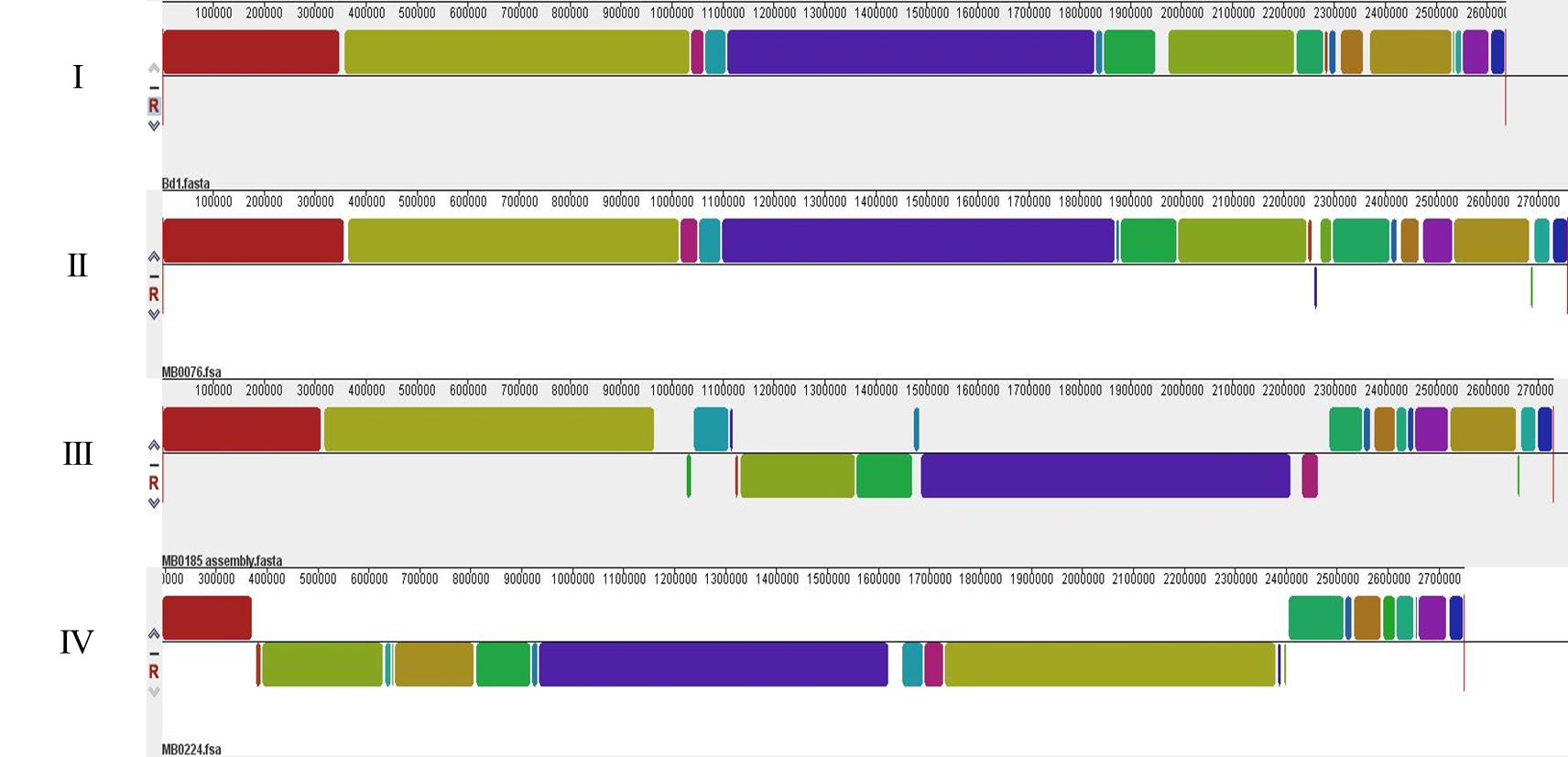
**

(B)

**
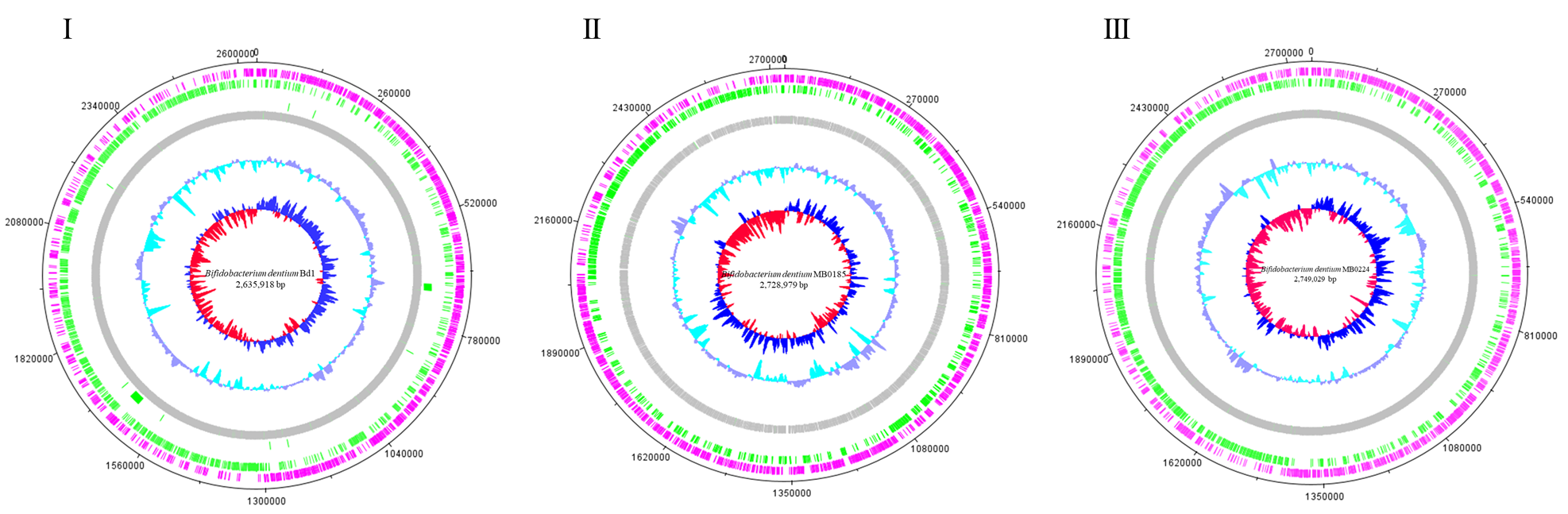
**

**Supplementary Figure 1**. **(A)** Genome-wide comparison of *B. dentium* Bd1 (I), *B. dentium* MB0076 (II), *B. dentium* MB0185 (III) and *B. dentium* MB0224 (IV) genomes. The progressive Mauve alignment shows the homologous blocks shared among the analyzed bifidobacteria genomes. The figure was generated using Mauve-Multiple Genome Alignment. **(B)** Genome atlas of *B. dentium* Bd1 (I), *B. dentium* MB0185 (II) and *B. dentium* MB0224 (III). From the outer circle to the inner circle, black indicates the total base pair, pink indicates forward strand, green indicates reverse strand, gray indicates a total number of contigs, violet and light blue indicate GCplot, and blue and red indicate GCskew.


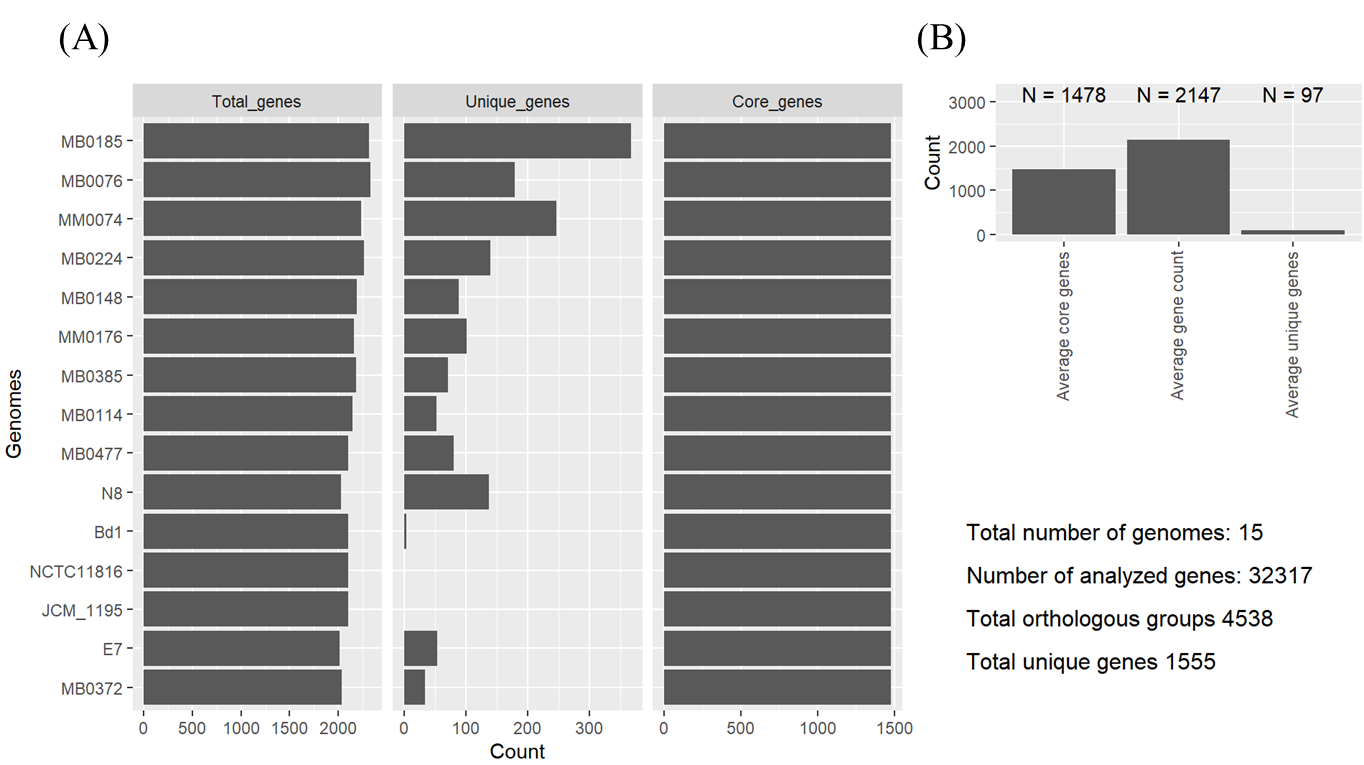


(B)

(A)

**Supplementary Figure 2.** Comparative genomics of 15 complete *B. dentium* genome sequences. **(A)** Representation of unique, core, and total genes present in the genome of individual *B. dentium* strain. **(B)** Bar chart representing the average gene count in each category.


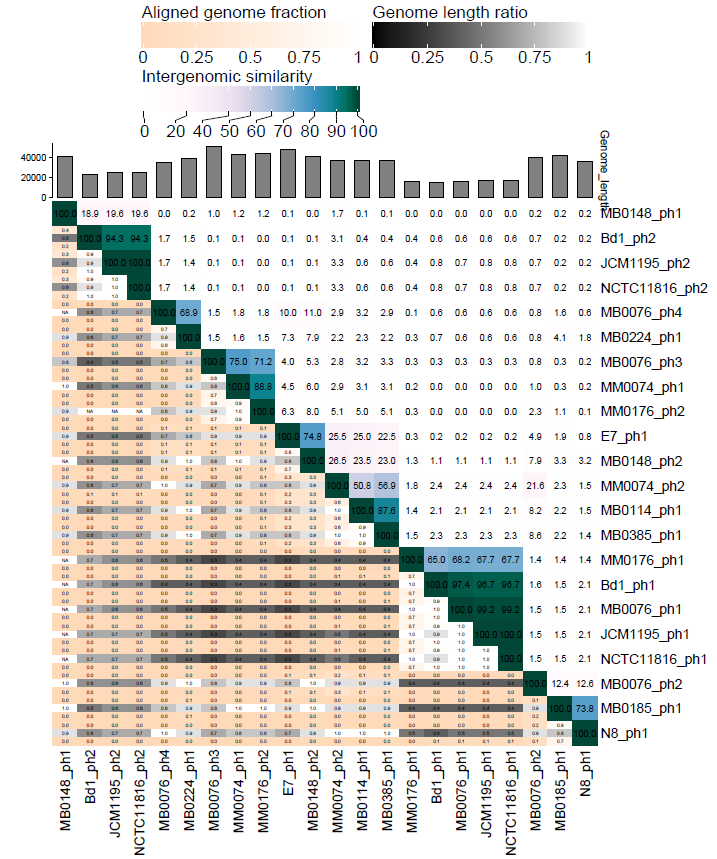


**Supplementary Figure 3.** Percentage sequence similarity between phage sequences present in 15 *B. dentium* genomes calculated using VIRIDIC. The horizontal and vertical coordinates indicate the phage sequence of the corresponding *B. dentium* strain.


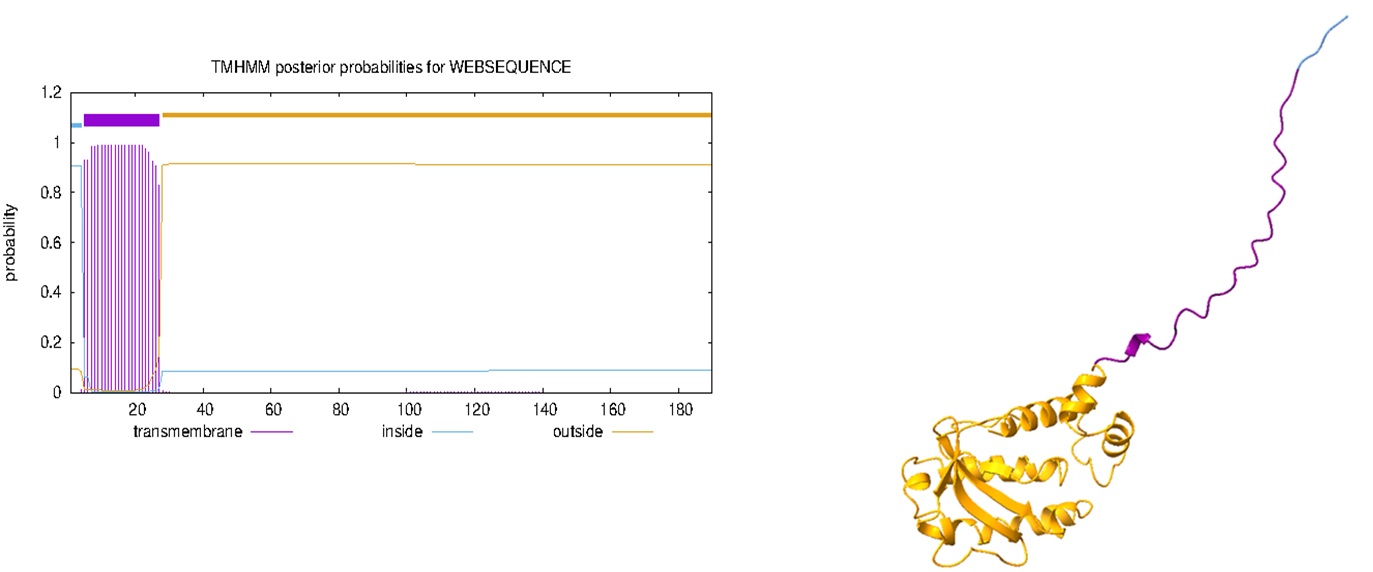


(B)

(A)

**Supplementary Figure 4.** *B. dentium* MB0148 propionicin-SM1-like bacteriocin predicted structure. **(A)** Topology prediction by TMHMM-2.0 server of *B. dentium* MB0148 propionicin-SM1-like protein sequence. **(B)** Ribbon model of the *B. dentium* MB0148 propionicin-SM1-like bacteriocin AlphaFold predicted structure is shown. The transmembrane domain, internal domain and outward domain are coloured in purple, cyan, and orange, respectively.


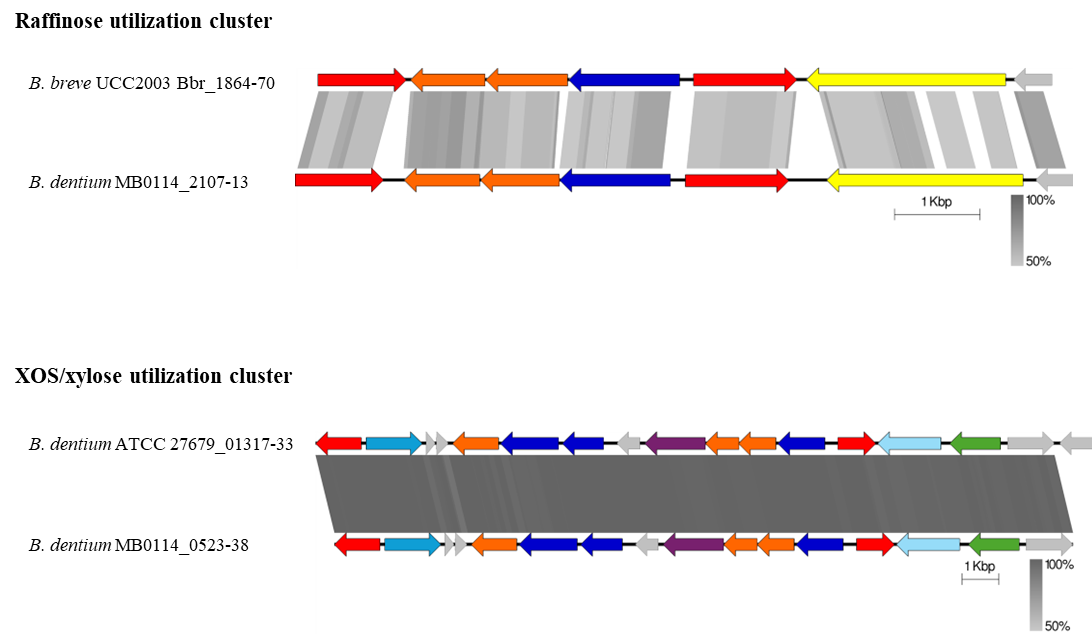


(B)

(A)


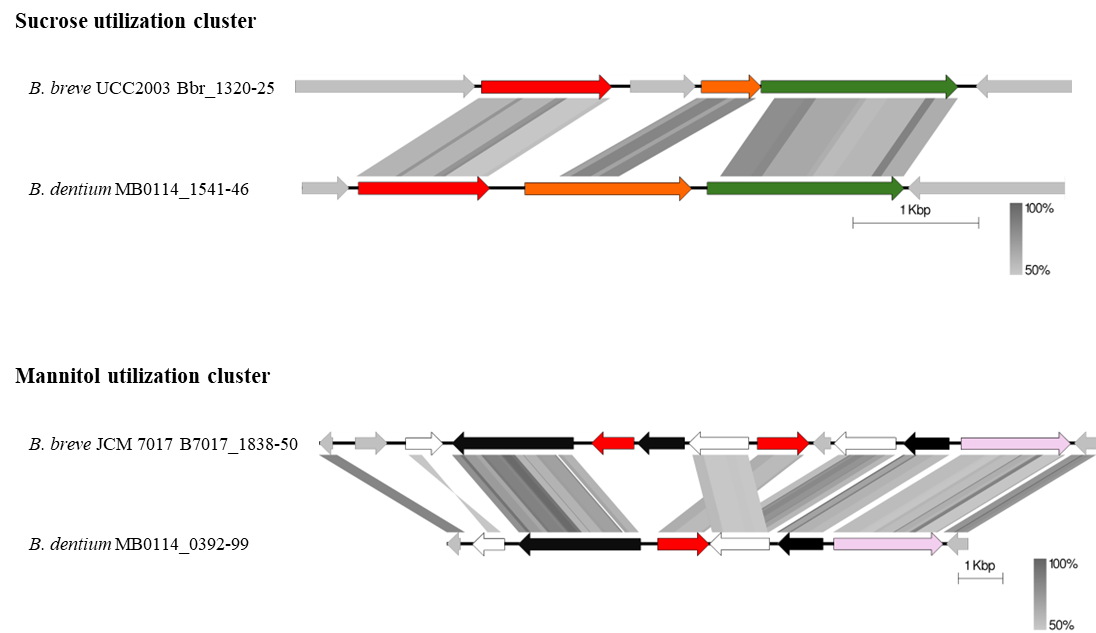

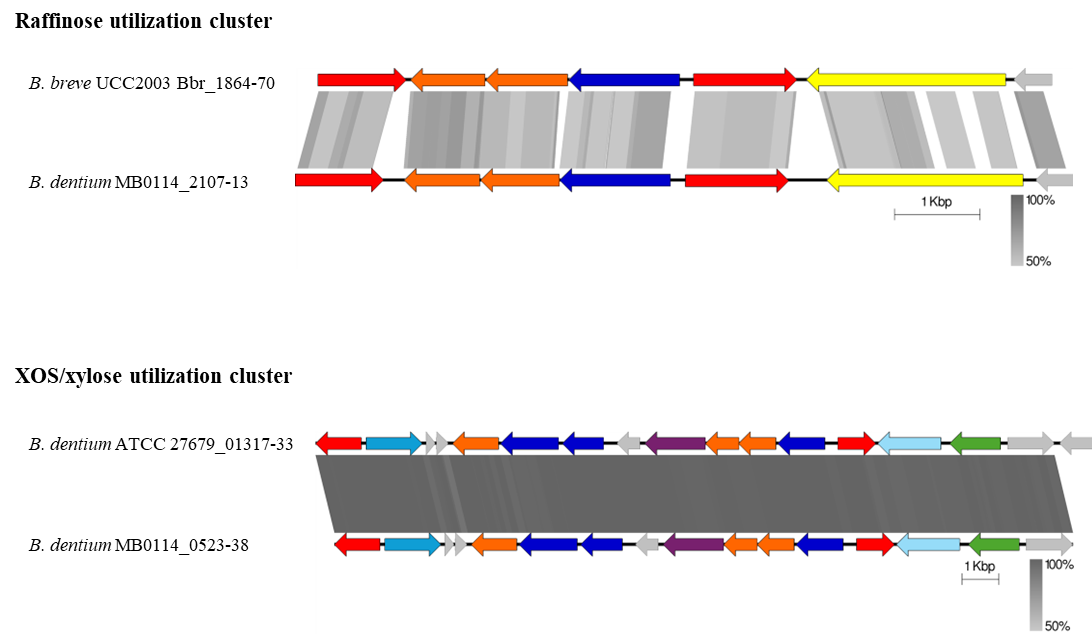

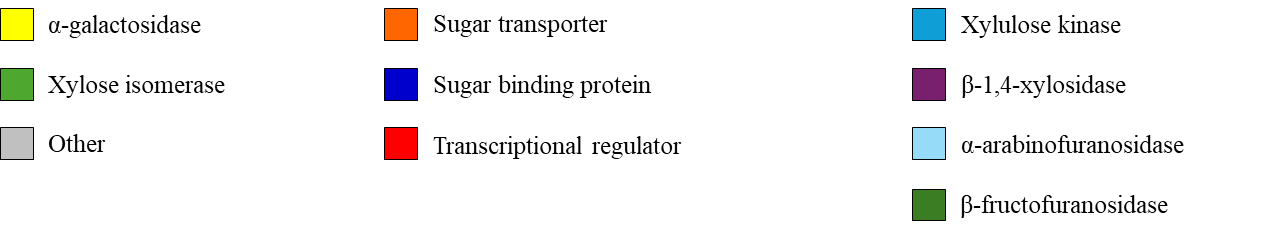


(C)

**Supplementary Figure 5.** Comparison of **(A)** raffinose, **(B)** sucrose**, (C)** XOS/xylose utilization clusters across bifidobacterial strains. The arrows represent coding sequences. Arrows pointing in the right direction are encoded on the forward strand, while arrows pointing in the left direction are encoded on the complementary strand. The size of the coding sequence is proportional to the length of the arrow. The predicted gene function is shown in the color legend. The percentage similarity between genes is indicated by the intensity of the grey connecting strand. The locus tag of the sequence is shown on the left-hand side.


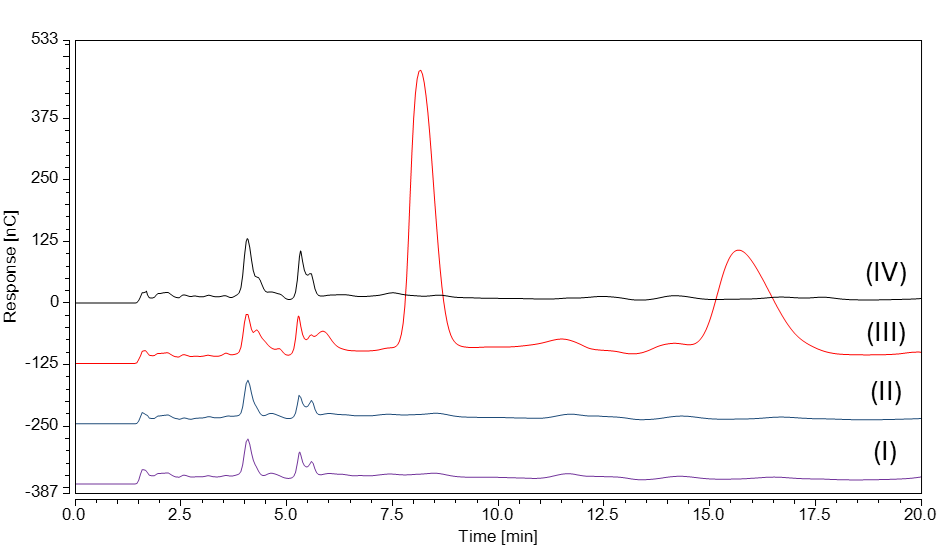


**Supplementary Figure 6.** HPAEC-PAD chromatogram profiles of *B. dentium* MM0074 and *B. dentium* MB0114 supernatants following 24 h of growth in 1% (v/v) XOS. (I) *B. dentium* MM0074 supernatant. (II) *B. dentium* MB0114 supernatant. (III) mMRS supplemented with 1% (v/v) XOS. (IV) mMRS.


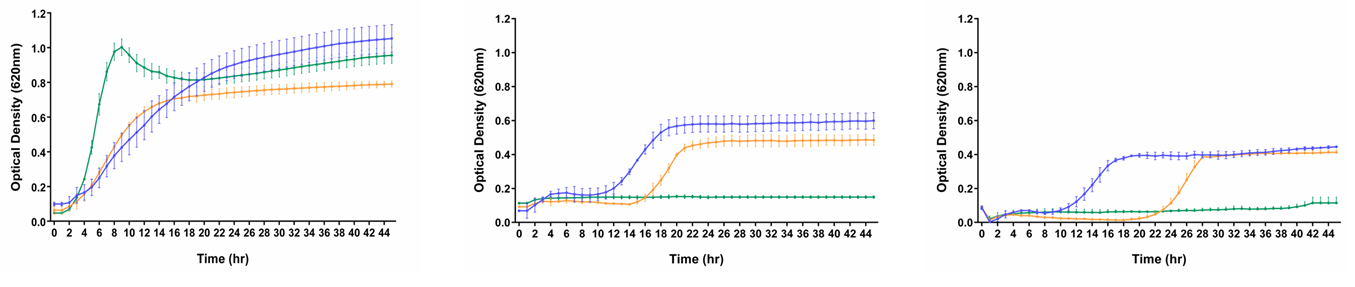

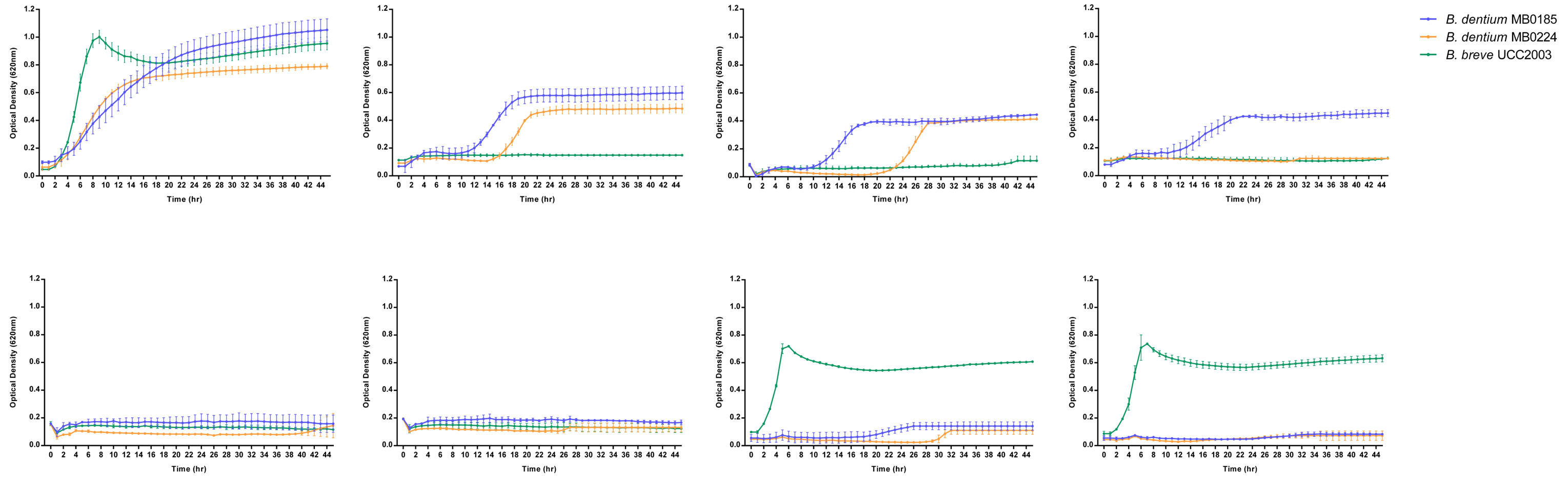


(C)

(D)

(A)

(B)


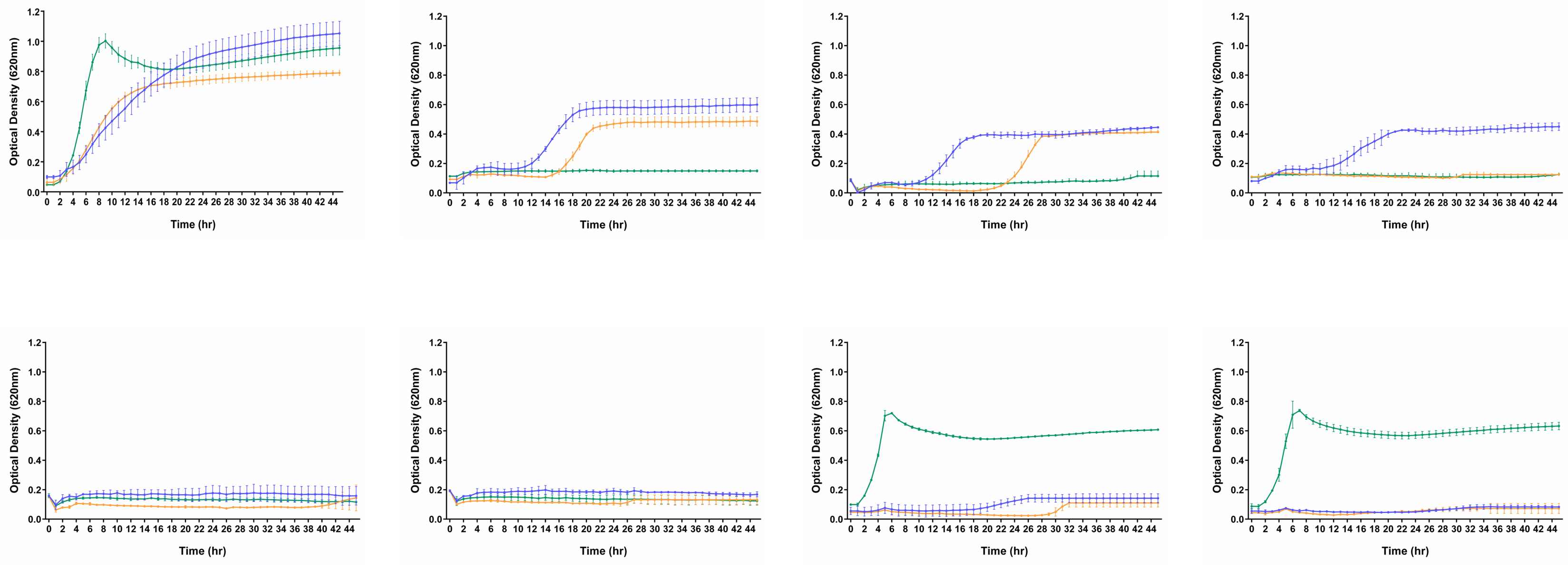


(F)

(E)


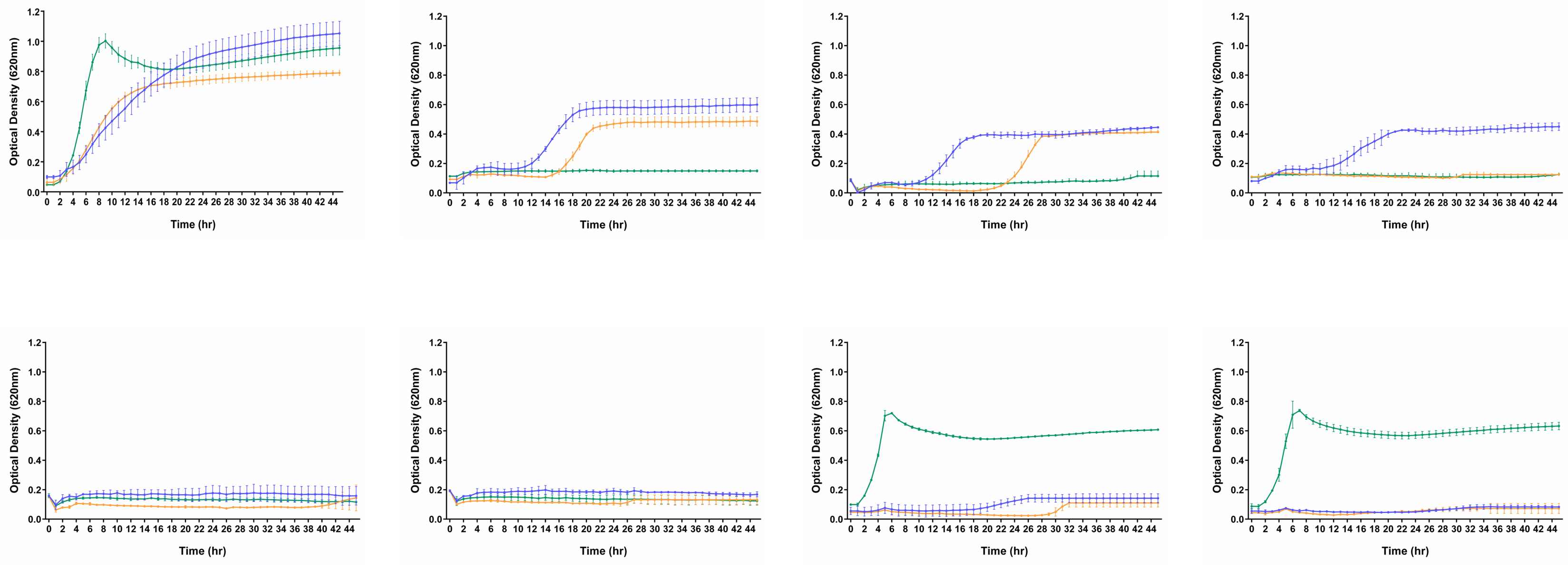


(H)

(G)


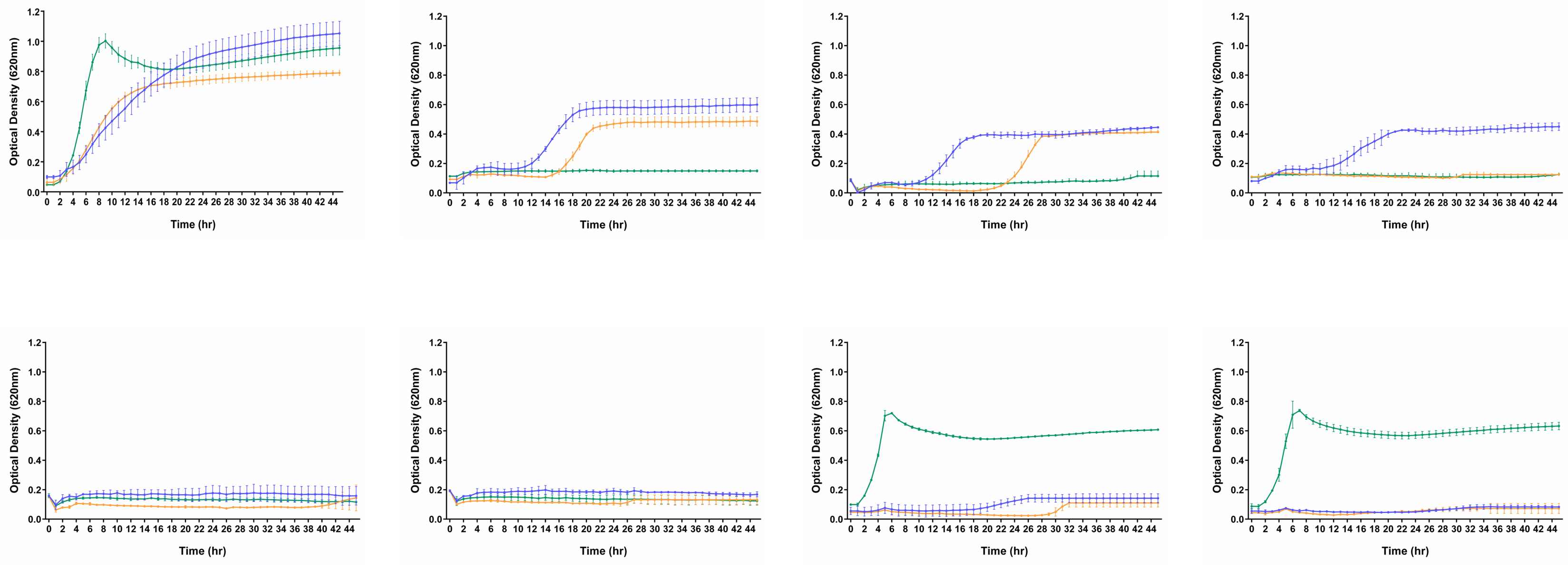


**Supplementary Figure 7.** Growth curves of *B. dentium* MB0185, *B. dentium* MB0224 and *B. breve* UCC2003 as an average of the optical density (OD) measures of duplicate cultures in mMRS supplemented with 1% (v/v) lactose **(A)** or 0.5% (v/v) 2’FL **(B),** 3FL **(C)**, DFL **(D)**, 6’SL **(E)**, 3’SL **(F)**, LNT **(G)** or LNnT **(H)** as the sole carbon source. Error bars represent the standard deviation. *B. breve* UCC2003 was used as positive control for growth on LNT and LNnT and as a negative control for the growth on 2’FL, 3FL, DFL, 6SL and 3’SL.


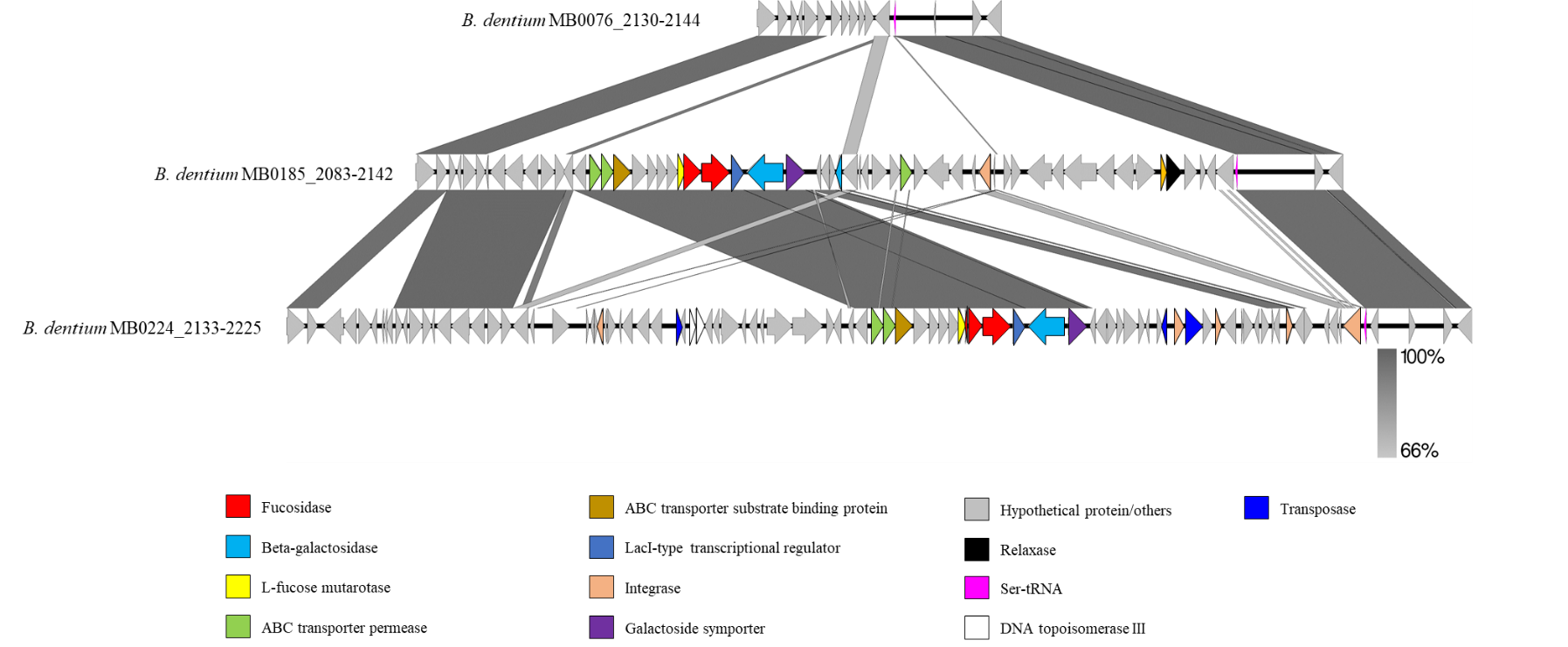


**Supplementary Figure 8.** Linear comparison of the genome region in which the fucosyllactose utilization locus is located across *Bifidobacterium dentium* strains. The comparison of gene clusters was created using Easyfig. The arrows represent coding sequences. Arrows pointing in the right direction are encoded on the forward strand, while arrows pointing in the left direction are encoded on the complementary strand. The size of the coding sequence is proportional to the length of the arrow. The predicted gene function is shown in the color legend. The percentage similarity between genes is indicated by the intensity of the grey connecting strand. The locus tag of the sequence is shown on the left-hand side.


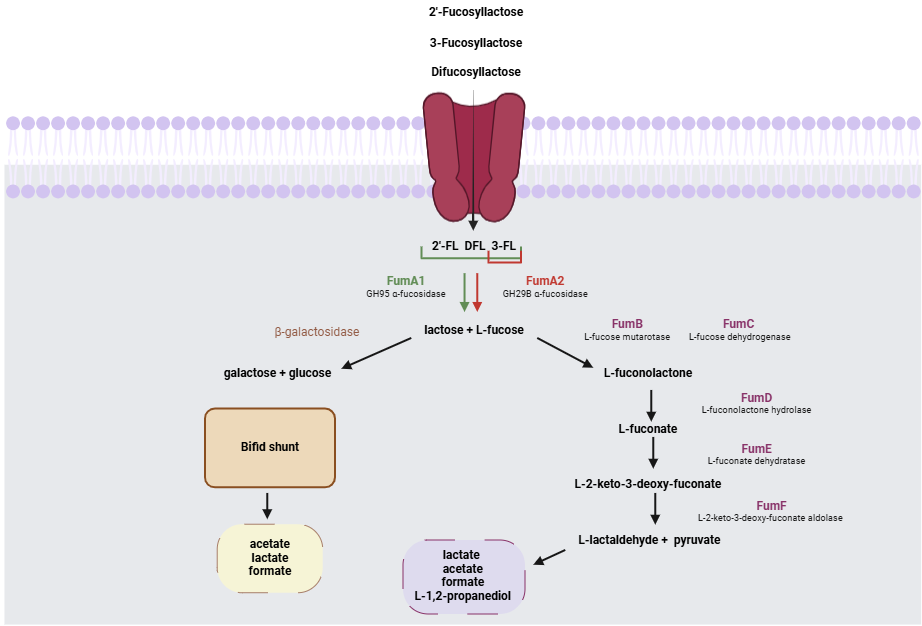


(A)

(B)


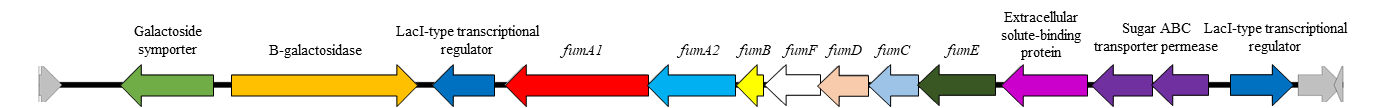


**Supplementary Figure 9. (A)** Schematic representation of the fucosyllactose utilization pathway and **(B)** the fucosyllactose cluster in *B. dentium* MB0185.


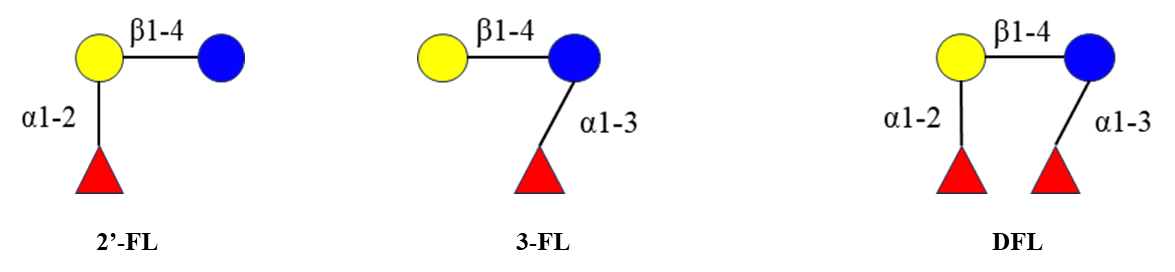


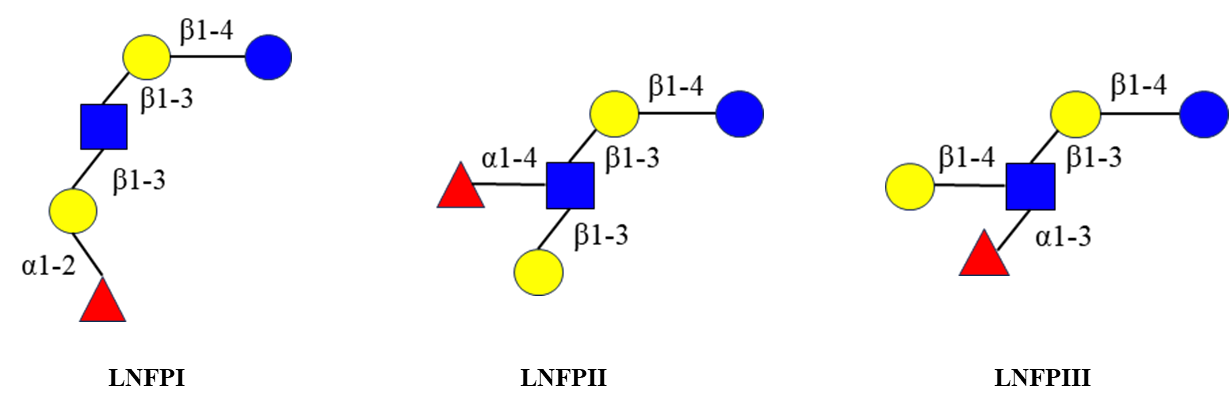


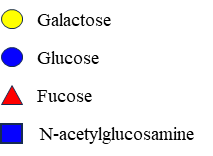


**Supplementary Figure 10.** Schematic structures of 2’fucosyllactose (2’FL), 3-fucosyllactose (3FL), difucosyllactose (DFL), lacto-N-fucopentaose I (LNFPI), lacto-N-fucopentaose II (LNFPII), lacto-N-fucopentaose III (LNFPIII).


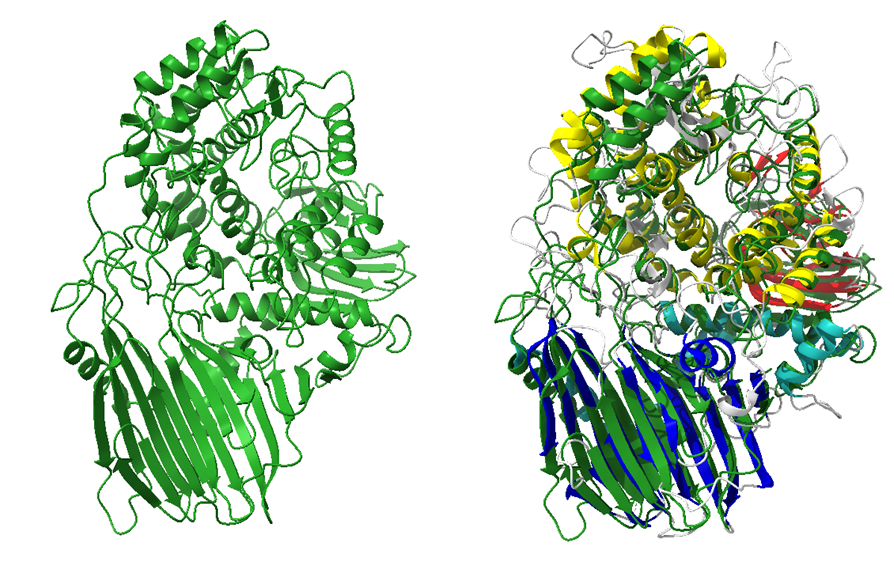

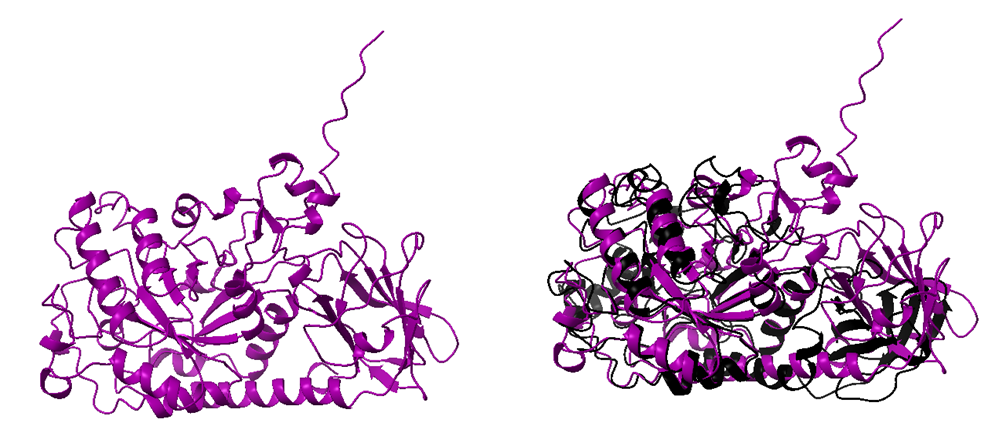


(B)

(A)

**Supplementary Figure 11.** AlphaFold predicted structure of *B. dentium* MB0185 FumA1 and FumA2. **(A)** Ribbon model of FumA1 predicted structure is shown. **(B)** Ribbon model of FumA2 predicted structure is shown.


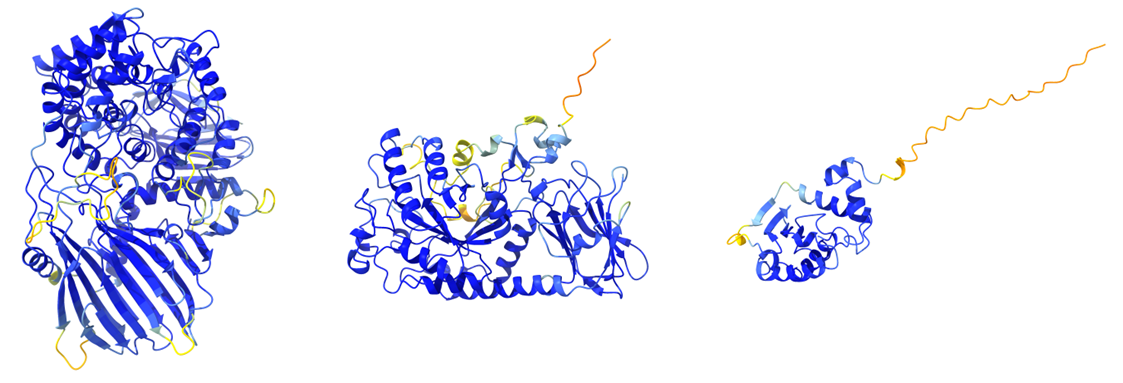


(C)

(D)

(B)

(A)

(F)

(E)


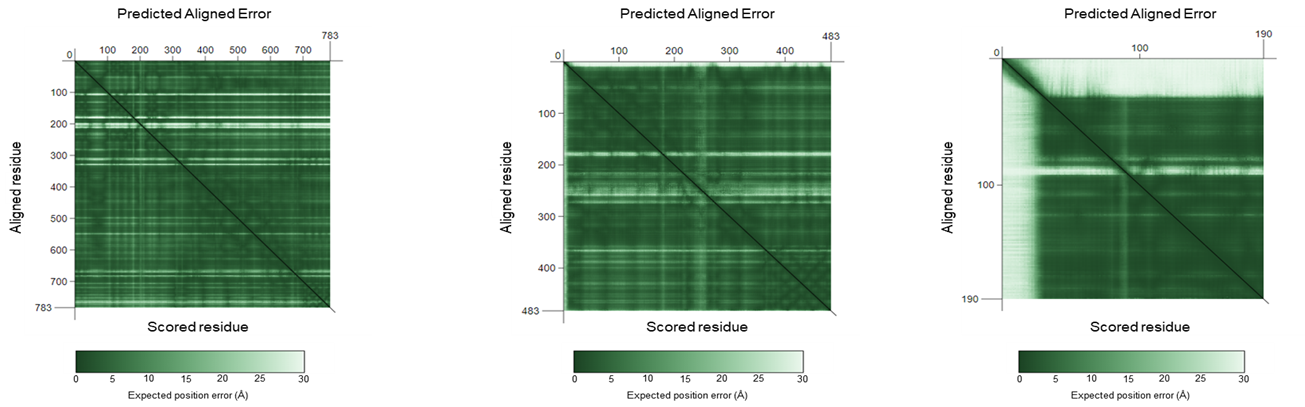


**Supplementary Figure 12.** AlphaFold predicted aligned errors (PAE) and predicted local distance difference (pLDDT) outputs. **(A)** *B. dentium* MB0185 FumA1 **(B)** *B. dentium* MB0185 FumA2 and **(C)** *B. dentium* MB0148 propionicin-SM1-like bacteriocin AlphaFold predictions colored by pLDDT. **(D)** FumA1 PAE graph. **(E)** FumA2 PAE graph. **(F)** propionicin-SM1-like bacteriocin PAE graph.

## Supplementary Tables

**Supplementary Table 1.** List of carbohydrates used for fermentation profiles.

| **Substrate** | **Source** |
| --- | --- |
| 2’-fucosyllactose | Glycom, Lyngby, Denmark |
| 3’-sialyllactose | DSM, Heerlen, Netherlands |
| 3-fucosyllactose | Glycom, Lyngby, Denmark |
| 6’-sialyllacose | DSM, Heerlen, Netherlands |
| Amylopectin from maize | Sigma-Aldrich, Germany |
| Arabinan | Megazyme, Ireland |
| Arabinogalactan | Lonza Inc., Switzerland |
| Arabinoxylan | Megazyme, Ireland |
| Arbutin | Sigma-Aldrich, Germany |
| Difucosyllactose | DSM, Heerlen, Netherlands |
| Galactan | Megazyme, Ireland |
| Galactose | Sigma-Aldrich, Germany |
| Glucose | Thermo Fisher Scientific, US |
| Glycogen from oyster | Sigma-Aldrich, Germany |
| Lacto-N-fucopentaose I | DSM, Heerlen, Netherlands |
| Lacto-N-fucopentaose II | DSM, Heerlen, Netherlands |
| Lacto-N-fucopentaose III | DSM, Heerlen, Netherlands |
| Lacto-N-neotetraose | Glycom, Lyngby, Denmark |
| Lacto-N-tetraose | Glycom, Lyngby, Denmark |
| Lactose | Sigma-Aldrich, Germany |
| L-Arabinose | Sigma-Aldrich, Germany |
| L-fucose | Sigma-Aldrich, Germany |
| Maltodextrin | Sigma-Aldrich, Germany |
| Maltose | Sigma-Aldrich, Germany |
| Mannitol | Sigma-Aldrich, Germany |
| Pullulan | Sigma-Aldrich, Germany |
| Raffinose | Sigma-Aldrich, Germany |
| Sorbitol | Sigma-Aldrich, Germany |
| Starch | Sigma-Aldrich, Germany |
| Sucrose | Thermo Fisher Scientific, US |
| Xylo-oligosaccharide (XOS) | Shandong Longlive Bio-technology Co., China |
| Xylan | Sigma-Aldrich, Germany |
| Xylose | Sigma-Aldrich, Germany |
| β-glucan (barley) | Megazyme, Ireland |

**Supplementary Table 2.** Oligonucleotides used in this thesis.

| **Name** | **Sequence (5’→3’)^a,b^** | **Relevant characteristics** |
| --- | --- | --- |
| GH95F | AGCAGCGCTAGCATGAAGCTTGTATTCCATGGAACC | NheI restriction site |
| GH95R | AGCAGCGCGGCCGCCTACCGATGGATGACACCTTC | NotI restriction site |
| GH29BF | AGCAGCGCTAGCATGAGCAATTTGATGAATACCAACG | NheI restriction site |
| GH29BR | AGCAGCGAATTCTCATGACAGCTCCTTTATGCG | EcoRI restriction site |

^a^. The restriction sites included in the oligonucleotides are underlined.

^b^. The promoters included in the oligonucleotides are in bold.

**Supplementary Table 3.** Mobilome features of *B. dentium* strains.

| **Genomes** | **Integrases/Transposases Number** | **Prophages Number** | **Mobile elements Number** | **RM systems Number** | **CRISPR-Cas Systems Number** |
| --- | --- | --- | --- | --- | --- |
| *B. dentium* Bd1 | 18 | 2 | 2 | 2  (0, 0, 0, 2)* | 2 |
| *B. dentium* JCM1195 | 18 | 2 | 2 | 3  (0, 1, 0, 2)* | 2 |
| *B. dentium* N8 | 18 | 1 | 2 | 2  (1, 1, 0, 0)* | 1 |
| *B. dentium* E7 | 28 | 1 | 2 | 3  (1, 1, 0, 1)* | 2 |
| *B. dentium* NCTC11816 | 18 | 2 | 2 | 2  (0, 1, 0, 1)* | 2 |
| *B. dentium* MM0074 | 30 | 2 | 2 | 0 | 1 |
| *B. dentium* MB0076 | 12 | 4 | 2 | 1  (1, 0, 0, 0)* | 1 |
| *B. dentium* MB0114 | 28 | 1 | 2 | 2  (1, 0, 1, 0)* | 2 |
| *B. dentium* MB0148 | 18 | 2 | 1 | 3  (1, 0, 2, 0)* | 1 |
| *B. dentium* MM0176 | 26 | 2 | 2 | 1  (1, 0, 0, 0)* | 1 |
| *B. dentium* MB0185 | 15 | 1 | 5 | 1  (1, 0, 0, 0)* | 1 |
| *B. dentium* MB0224 | 24 | 1 | 4 | 2  (2, 0, 0, 0)* | 1 |
| *B. dentium* MB0372 | 26 | 0 | 4 | 0 | 1 |
| *B. dentium* MB0385 | 13 | 1 | 2 | 0 | 1 |

(a, b, c, d)*. The number of type I (a), type II (b), type III (c) and type IV (d) RM systems are reported.

**Supplementary Table 4.** G-tract mapping in sortase-dependent pilus genes in 10 *B. dentium* strains. Columns from left to right indicate the name of the *B. dentium* strain, the number of sortase-dependent pilus cluster(s), the locus of clusters containing a G-tract, their specific nt length, the corresponding genomic coordinates and chromosomal DNA strand.

| **Strain** | **Sortase pilus clusters (total)** | **PolyG pilus locus** | **PolyG length (bp)** | **PolyG position** | **Strand** |
| --- | --- | --- | --- | --- | --- |
| Bd1 | 7 | BDP_RS02615-25 | 11 | 629857-629867 | + |
|  |  | BDP_RS09000-10 | 13 | 2145404-2145416 | - |
|  |  | BDP_RS10435-55 | 9 | 2516862-2516870 | - |
| E7 | 5 | J7M36_RS00690-05 | 11 | 176976-176986 | + |
|  |  | J7M36_RS10540-50 | 10 | 633200-633209 | + |
| JCM_1195 | 7 | BBDE_RS00665-80 | 11 | 173280-173290 | + |
|  |  | BBDE_RS02620-35 | 10 | 629859-629868 | + |
|  |  | BBDE_RS09005-15 | 15 | 2144869-2144883 | - |
|  |  | BBDE_RS10435-55 | 9 | 2516246-2516254 | - |
| N8 | 5 | J7M35_RS00815-30 | 11 | 197915-197925 | + |
|  |  | J7M35_RS08435-50 | 10 | 2034919-2034928 | - |
|  |  | J7M35_RS10275-90 | 11 | 2468221-2468231 | - |
| NCTC11816 | 7 | EL182_RS00695-10 | 10 | 173280-173289 | + |
|  |  | EL182_RS02695-10 | 10 | 629858-629867 | + |
|  |  | EL182_RS09200-10 | 13 | 2144867-2144879 | - |
|  |  | EL182_RS10675-95 | 9 | 2516324-2516332 | - |
| MB0076 | 7 | MB0076_0131-34 | 11 | 172730-172740 | + |
|  |  | MB0076_1873-75 | 12 | 2174135-2174146 | - |
|  |  | MB0076_1960-62 | 13 | 2266159-2266171 | - |
|  |  | MB0076_2277-79 | 10 | 2671363-2671372 | - |
| MB0114 | 5 | MB0114_0130-33 | 10 | 171812-171821 | + |
| MB0148 | 8 | MB0148_0129-33 | 10 | 171910-171919 | + |
|  |  | MB0148_1763-66 | 12 | 2115034-2115045 | - |
|  |  | MB0148_1846-48 | 14 | 2205982-2205995 | - |
|  |  | MB0148_2150-54 | 11 | 2604276-2604286 | - |
| MB0185 | 5 | MB0185_0109-0113 | 10 | 140883-140892 | + |
|  |  | MB0185_0962-64 | 21 | 1115130-1115150 | + |
|  |  | MB0185_1057-60 | 12 | 1217542-1217553 | + |
| MB0224 | 8 | MB0224_0155-59 | 11 | 195178-195188 | + |
|  |  | MB0224_0374-77 | 11 | 463146-463157 | + |
|  |  | MB0224_0523-27 | 12 | 665047-665058 | + |
|  |  | MB0224_1977-79 | 11 | 2388742-2388752 | - |
| MB0372 | 5 | MB0372_0143-47 | 10 | 183033-183042 | + |
| MB0385 | 8 | MB0385_0120-24 | 9 | 160188-160196 | + |
|  |  | MB0385_1728-31 | 10 | 2070285-2070294 | - |
|  |  | MB0385_1813-15 | 12 | 2163080-2163091 | - |
|  |  | MB0385_2097-00 | 10 | 2511202-2511211 | - |
| MB0477 | 7 | MB0447_0137-41 | 9 | 178977-178985 | + |
|  |  | MB0447_1735-39 | 10 | 2092646-2092655 | - |
|  |  | MB0447_1923-27 | 12 | 2352655-2352666 | - |
| MM0074 | 6 | MM0074_0134-38 | 10 | 175437-175446 | + |
| MM0176 | 7 | MM0176_0132-36 | 10 | 176780-176789 | + |
|  |  | MM0176_1797-00 | 11 | 2122626-2122636 | - |
|  |  | MM0176_2073-77 | 11 | 2472154-2472164 | - |
